# Type IV Pili of Streptococcus sanguinis Contribute to Pathogenesis in Experimental Infective Endocarditis

**DOI:** 10.1128/Spectrum.01752-21

**Published:** 2021-11-10

**Authors:** Anthony M. Martini, Bridget S. Moricz, Laurel J. Woods, Bradley D. Jones

**Affiliations:** a Department of Microbiology and Immunology, University of Iowa Carver College of Medicine, Iowa City, Iowa, USA; b Graduate Program in Genetics, University of Iowa, Iowa City, Iowa, USA; Emory University School of Medicine

**Keywords:** *Streptococcus sanguinis*, bacterial pathogenesis, infective endocarditis, motility, platelet adherence, platelet aggregation, rabbit model, type IV pili

## Abstract

Streptococcus sanguinis is a common cause of infective endocarditis (IE). Efforts by research groups are aimed at identifying and characterizing virulence factors that contribute to the ability of this organism to cause IE. This Gram-positive pathogen causes heart infection by gaining access to the bloodstream, adhering to host extracellular matrix protein and/or platelets, colonizing the aortic endothelium, and incorporating itself into the aortic vegetation. While many virulence factors have been reported to contribute to the ability of S. sanguinis to cause IE, it is noteworthy that type IV pili (T4P) have not been described to be a virulence factor in this organism, although S. sanguinis strains typically encode these pili. Type IV pili are molecular machines that are capable of mediating diverse virulence functions and surface motility. T4P have been shown to mediate twitching motility in some strains of S. sanguinis, although in most strains it has been difficult to detect twitching motility. While we found that T4P are dispensable for direct *in vitro* platelet binding and aggregation phenotypes, we show that they are critical to the development of platelet-dependent biofilms representative of the cardiac vegetation. We also observed that T4P are required for *in vitro* invasion of S. sanguinis into human aortic endothelial cells, which indicates that S. sanguinis may use T4P to take advantage of an intracellular niche during infection. Importantly, we show that T4P of S. sanguinis are critical to disease progression (vegetation development) in a native valve IE rabbit model. The results presented here expand our understanding of IE caused by S. sanguinis and identify T4P as an important virulence factor for this pathogen.

**IMPORTANCE** This work provides evidence that type IV pili produced by Streptococcus sanguinis SK36 are critical to the ability of these bacteria to attach to and colonize the aortic heart valve (endocarditis). We found that an S. sanguinis type IV pili mutant strain was defective in causing platelet-dependent aggregation in a 24-h infection assay but not in a 1-h platelet aggregation assay, suggesting that the type IV pili act at later stages of vegetation development. In a rabbit model of disease, a T4P mutant strain does not develop mature vegetations that form on the heart, indicating that this virulence factor is critical to disease and could be a target for IE therapy.

## INTRODUCTION

Infective endocarditis (IE) is a microbial infection of the endocardium and is predominantly caused by Gram-positive bacteria of the genera Streptococcus, Staphylococcus, and *Enterococcus* (1). Colonization of the endocardium results in the development of a vegetative mass composed of microorganisms and host factors, which is typically located on the heart valve. In both developed and developing nations, morbidity and mortality associated with IE remains high despite decades of medical advancement (2–4). Clinical management of IE is complicated by the range of predisposing conditions and variety of etiological pathogens that cause this disease, while nonspecific symptoms often delay diagnosis (5, 6). Although the advent of surgical intervention has significantly improved patient outcomes, effective therapeutic and diagnostic tools are still unavailable in developing regions of the world. As little progress has been made toward prevention of this life-threatening infection, it continues to be a high priority to develop effective therapies for IE (7, 8).

Streptococcus sanguinis is a pioneering oral commensal associated with good oral health, but it also behaves as an opportunistic pathogen when the organisms gain access to the bloodstream. Along with other oral streptococci, these microorganisms are responsible for an estimated 20% of IE cases worldwide (1, 9). In developing areas of the world, where rheumatic heart disease is a common predisposing condition, these organisms are particularly prevalent and often difficult to diagnose and treat (3). The ability of oral streptococci to cause IE is associated with their capacity to bind and activate platelets, adhere to host tissues and extracellular factors, and to synthesize exopolysaccharides (EPS) (10–15). While a subset of pathogenic determinants is shared among species, it is clear that different species possess unique factors that confer distinct pathogenic potentials (16). Notably, S. sanguinis has been identified as one of the most common species in streptococcal IE (17–19).

A unique characteristic of S. sanguinis, in contrast to other oral streptococci, is that they can exhibit twitching motility on agar plates mediated by polar fimbriae (20). This motility phenotype was originally associated with agglutination of red blood cells, but it has no impact on competence or transformation (20–22). As in other bacterial species, twitching motility is mediated by type IV pili (T4P), which are long, surface-exposed filaments composed of polymerized pilin subunits (23–25). Aside from mediating motility, T4P function as virulence factors in many other bacteria where they facilitate host cell interactions and contribute to biofilm formation, as well as activation of inflammatory pathways (26–29).

**FIG 1:**
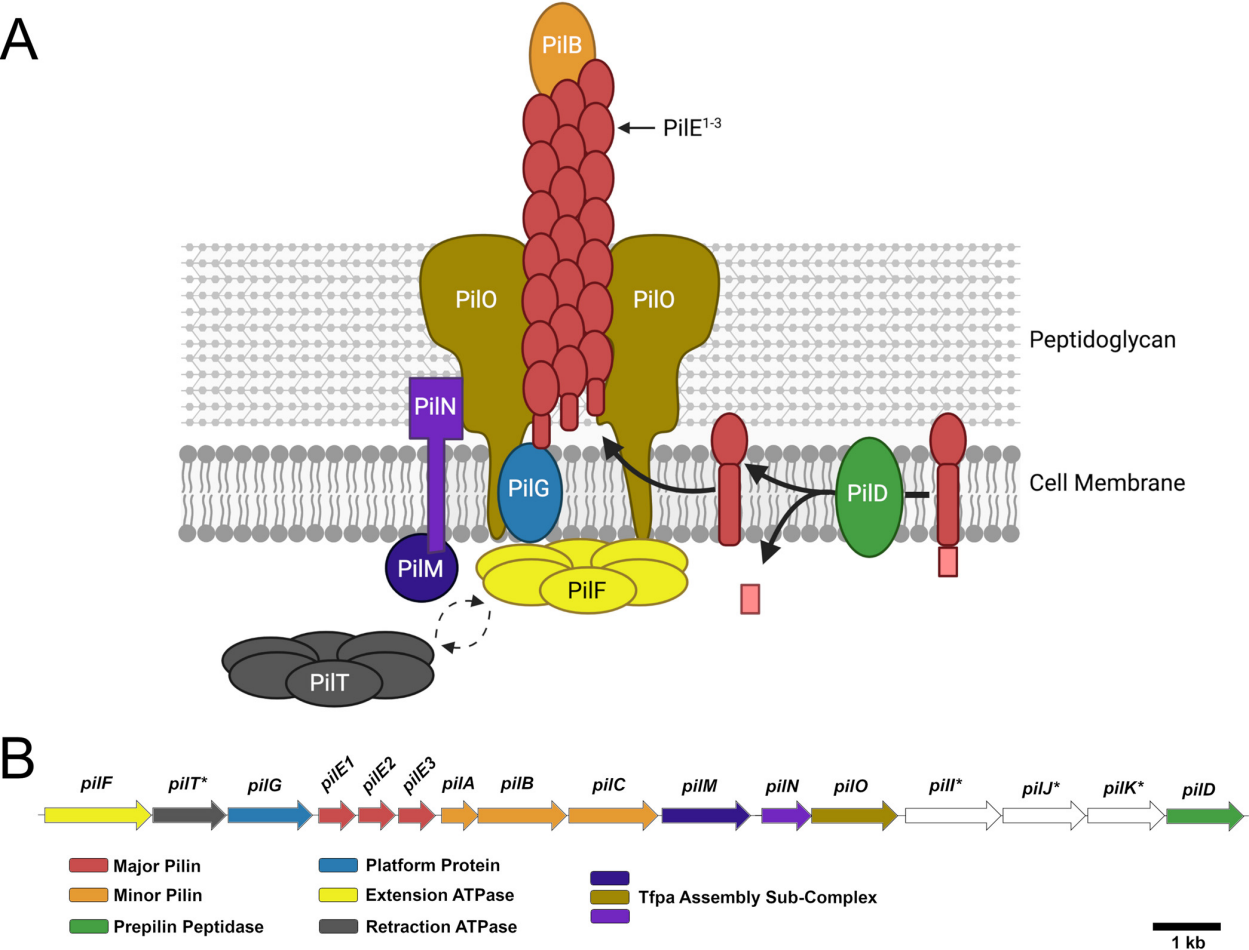
Type IV pili in S. sanguinis SK36. (A) Model of T4P filament assembly in S. sanguinis, with PilB localized to the end of the filament based on structural modeling by Raynaud et al. (66). PilACIJK are not shown due to unknown function or localization. Image created with BioRender. (B) Genes involved in T4P biogenesis in monoderm bacteria, adapted from Pelicic (55). Known or predicted functions are indicated below the operon. Genes of the same color are predicted to encode proteins of similar function, while those colored in white have no identified homologous counterparts in other species. An asterisk indicates genes dispensable for filament assembly but essential for functional motility.

Recently, the molecular machinery responsible for motility in S. sanguinis was characterized, and nearly all sequenced S. sanguinis species have been found to carry genes encoding T4P (30). This work identified the presence of both known molecular components previously characterized in other T4P systems and novel features of the S. sanguinis system. Figure 1 shows a schematic of T4P assembly (Fig. 1A) and the T4P gene locus (Fig. 1B). A retraction ATPase, PilT, mediates surface motility by depolymerizing pili filaments and generating tension force (23). Encoded along with PilT are several proteins conserved in T4P biosynthesis. These include two or three major pilus subunits (PilE1, PilE2, and PilE3) as well as three minor subunits (PilA, PilB, PilC), which are processed by PilD, a prepilin peptidase. Filament assembly is performed by the traffic ATPase, PilF, via polymerization of the PilE subunits. S. sanguinis mutants in *pilF*, *pilD*, or a double mutant in both *pilE* genes are nonpiliated; interestingly, mutants in only one of the *pilE* genes (E1 or E2 in strain 2908) produced filaments composed of the other PilE isotype (30). Some T4P genes are dispensable for surface expression of T4P, although mutants in those genes do not exhibit macroscopic twitching motility; this includes strains with mutations in *pilT*, *pilI*, *pilJ*, and *pilK*. The latter three genes encode proteins of unknown function and have not been described for T4P in Gram-negative species.

Given the relatively recent characterization of this T4P system in S. sanguinis, few contemporary studies have evaluated the contribution of T4P to virulence in a disease model. Notably, a recent publication reported the contribution of T4P to epithelial cell adherence and *in vitro* biofilm formation (31). The authors additionally describe carbon catabolite repression (CcpA)-dependent regulation of the T4P operon and demonstrate the production of type IV filaments. Another study described regulation of the retraction ATPase, *pilT*, via a specific CiaRH-controlled small RNA, which also modulated biofilm formation (32). Because of the relative lack of understanding of T4P in Gram-positive species, our primary goal in these studies was to characterize the virulence potential of T4P in S. sanguinis, and we hypothesized that their activity would be essential to disseminated disease such as IE. While these prior studies suggested a functional role for T4P in S. sanguinis, it is interesting to note that the two strains used, SK36 and ATCC 10556, respectively, had previously been determined to be nonmotile and nonpiliated. Indeed, early studies observed that some S. sanguinis encoding T4P nonetheless failed to exhibit twitching motility (20, 21), and a previously published effort to isolate pili from strain SK36 failed (30). Considering these disparities, an additional goal of our work was to evaluate whether T4P are either nonfunctional or differentially regulated in some S. sanguinis strains, most notably the commonly studied IE strain, SK36.

In this report, we confirm that T4P are produced by SK36 and that they can mediate platelet-bacteria interactions (platelet-dependent biofilm formation) at later stages of aggregate formation. We also observed that T4P are required for entry into aortic endothelial cells, which could promote inflammation and allow long-term persistence of the bacteria at the site of primary endothelial damage during infection. Importantly, we provide clear evidence that the T4P of S. sanguinis are essential to vegetation development and pathogenesis in a rabbit model of native valve infective endocarditis. Further, we present evidence that the *in vitro* platelet interactions that we observe are mediated by the metal-ion-dependent adhesion site (MIDAS) domain of PilB. Our results indicate that S. sanguinis T4P are an important virulence factor in IE.

## RESULTS

### S. sanguinis SK36 produces detectable type IV pilus proteins.

Almost 50 years ago, published observations of twitching motility in S. sanguinis described heterogeneity among strains in their ability to exhibit colony spreading and produce the associated filaments (20, 21). Recently published work found that S. sanguinis strains SK36 and ATCC 10556 were both nonmotile in surface motility assays (30, 32), and efforts to detect pili on the surface of SK36 were unsuccessful (30). Although T4P could not be identified on the surface of SK36 in these studies, other work using immunogold surface labeling did detect the presence of T4P on SK36 (31).

**FIG 2:**
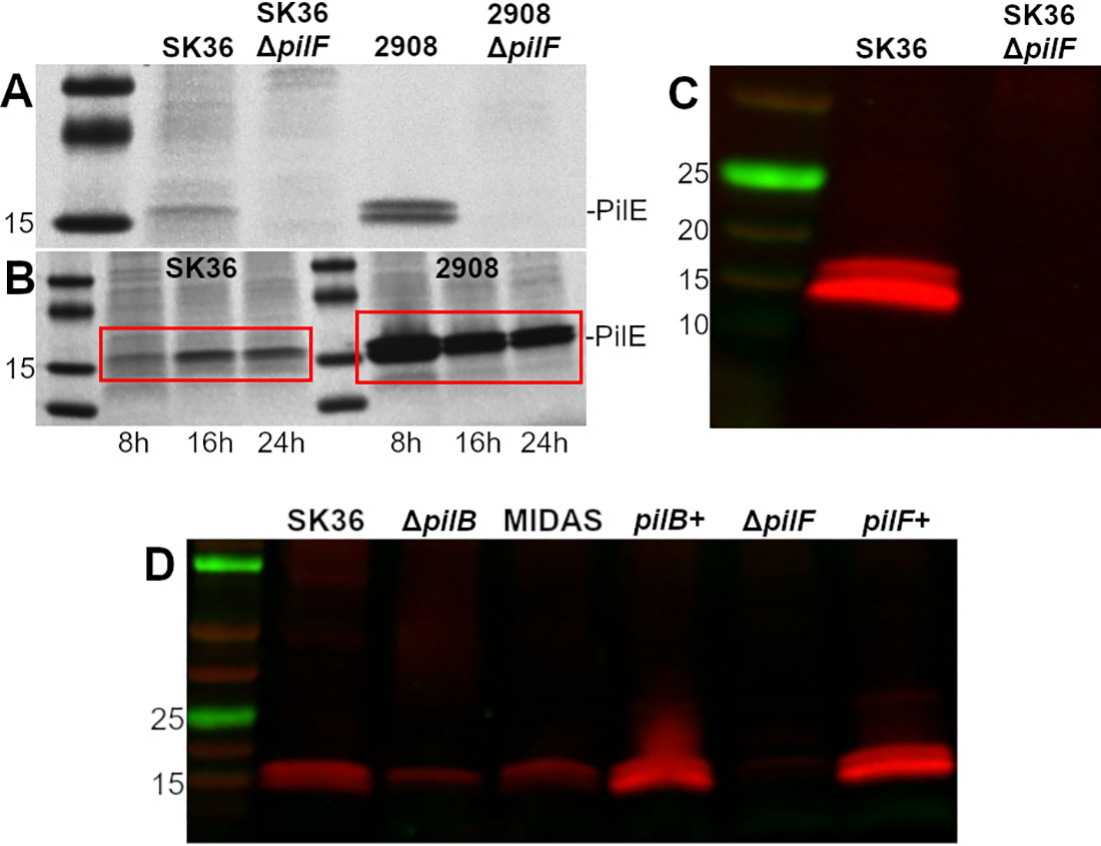
S. sanguinis SK36 produces T4P regulated by growth phase and detectable by immunoblotting. (A) R250 Coomassie staining for sheared T4P from S. sanguinis strains SK36 and 2908 and their isogenic Δ*pilF* mutants grown for 24 h in TH. (B) Colloidal G250 Coomassie staining of sheared SK36 and 2908 pili at different time points (red box). The 8-h time point denotes an OD_600_ of ∼1.0 (generally 8 h), while 16- and 24-h time points were normalized to an OD_600_ of 1.0 at the specified time postinoculation and then pelleted to ensure a comparable number of bacteria were processed. (C) Immunoblot of sheared pili (PilE) from SK36 and the Δ*pilF* mutant at 24 h. (D) Immunoblot of sheared pili from Δ*pilB*, MIDAS, and complementation strains. Results shown are representative of at least two independent experiments. Molecular weights (kDa) are indicated to the left of each image.

Consistent with these results, our group, using PAGE and Coomassie staining, identified pilin subunits produced by S. sanguinis SK36 when grown to late stationary phase (Fig. 2A). Pili were not detectable in an isogenic SK36 Δ*pilF* mutant, which is predicted to be nonpiliated due to the inability of this mutant to extend pilin outside of the cell, when grown under the same conditions as the wild-type (WT) strain. Due to the relatively low quantity of pili isolated from SK36 compared to that of a type IV pili-positive control S. sanguinis 2908 strain, we utilized a colloidal staining approach to increase sensitivity. Under these conditions, we could detect pili in late exponential/early stationary phase (optical density at 600 nm [OD_600_] = ∼1.0) but still observed an increased concentration of pili after more than 16 h of growth into stationary phase (OD_600_ = ∼1.3). (Fig. 2B). In contrast to strain SK36, the previously characterized strain 2908 produced high quantities of T4P regardless of growth phase. To facilitate future investigations, we commissioned the production of peptide-generated, polyclonal antibodies targeted toward the major pilin subunits of S. sanguinis SK36. Immune serum targeted toward PilE2 produced the strongest response (data not shown) and effectively distinguished between the WT and nonpiliated Δ*pilF* mutant by Western blotting after 24 h of growth (Fig. 2C). Preimmune serum did not produce a response (data not shown). Using a chromosomal complementation method to account for possible off-target mutations, we also demonstrate that reintroduction of the *pilF* gene via genomic crossover into the Δ*pilF* mutant restores T4P production (Fig. 2D).

### Strain SK36 exhibits twitching motility on blood agar.

**FIG 3:**
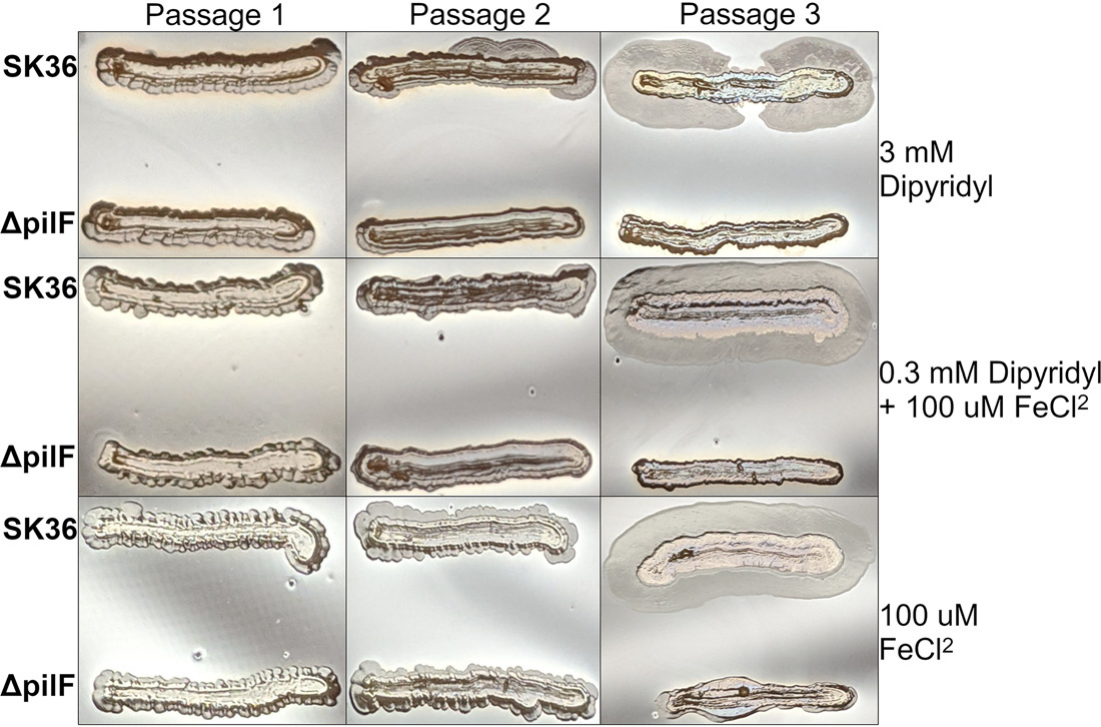
Twitching motility of SK36 when cultured on blood agar in the presence of excess (100 μM FeCl_2_), standard (0.3 mM dipyridyl + 100 μM FeCl_2_), or limited iron (3 mM dipyridyl). Plates were made with 5% sheep blood and 80% Levinthal’s medium base. Passaging of cultures was performed by scraping the outer edge of the colony with a sterile toothpick and restreaking organisms for isolation. Each passage was incubated for 4 days at 37°C in a candle jar containing sterile water at the bottom to provide humidity. Results shown are representative of at least three independent experiments.

A recently published report described the production and detection of T4P in SK36, but the authors were unable to detect twitching motility under any tested condition (31). Based on observations in our laboratory, and consistent with the role of twitching-based motility in other bacteria, we hypothesized that S. sanguinis T4P-mediated motility is repressed, or uninduced, by laboratory plating conditions on standard media. After reviewing conditions used in the earliest observations of S. sanguinis twitching motility (20, 21, 33, 34), we developed a microaerobic surface agar assay using Levinthal’s medium base supplemented with 5% defibrinated sheep blood. These assay conditions reproducibly produced twitching motility in strain 2908 (data not shown), which is known to display twitching motility (30). The development of twitching motility in SK36 appears reliably after one to two passages on plates and was not dependent on iron concentration (Fig. 3). Iron was investigated as a regulatory signal since preliminary work suggested it might play a role. Importantly, twitching motility was never observed in the isogenic Δ*pilF* mutant even after three passages and extended growth time (data not shown). Complementation by reintegration of the *pilF* gene into the chromosome of the Δ*pilF* mutant resulted in development of twitching motility similar to the parent strain (see Fig. S1 in the supplemental material).

Additionally, we wished to confirm that genetic disruption of SK36 T4P would abrogate twitching motility observed in the previous experiments. To this end, we isolated single colonies of motile SK36 WT described in Fig. 3 and transformed the Δ*pilF* mutation into these strains. Ten colonies across three independent experiments were mutagenized, and all 10 colonies were observed to lose twitching motility under permissive plate conditions; representative images from two independent colonies are shown in Fig. S2 in the supplemental material. Together, these data indicate that S. sanguinis SK36 produces functional T4P capable of mediating surface motility and that this phenotype is lost in a Δ*pilF* mutant. Further, the development of twitching motility can be restored by chromosomal complementation. This corroborates recent work identifying T4P on the surface of SK36 and expands this observation by demonstrating T4P function using a classical *in vitro* phenotype (31).

### Type IV pili are dispensable for *in vitro* platelet adherence and aggregation.

**FIG 4:**
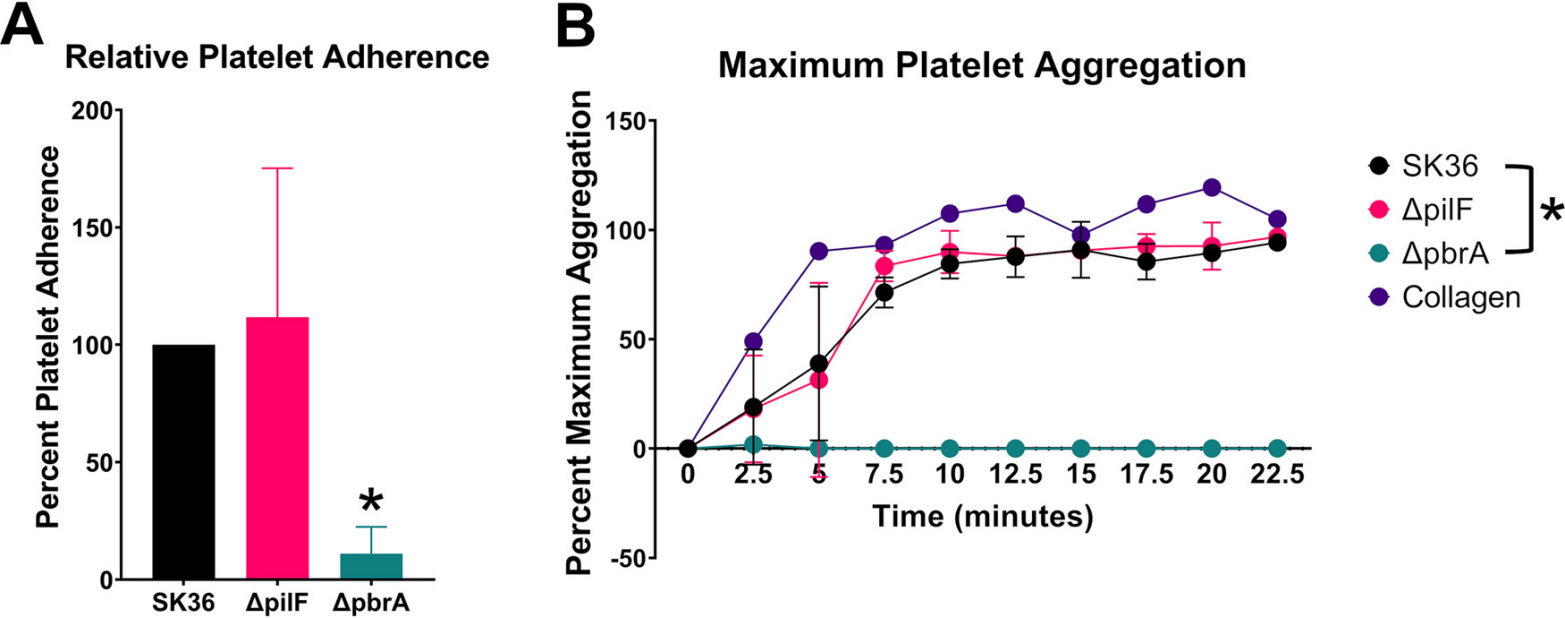
T4P are not required for *in vitro* platelet binding or *in vitro* platelet aggregation. (A) Binding of immobilized bacteria to human platelets. Bacteria were first adhered to the tissue culture well surface, wells were blocked with BSA, and human platelets were added to the wells to allow binding to the bacteria. Adherence shown is relative to WT and is correlated with acid phosphatase released from attached platelets in an acid phosphatase assay, with pNPP as substrate. (B) Aggregation of human platelets was measured by light transmission aggregometry using a modified microtiter plate assay. The Δ*pbrA* strain was included in both sets of experiments as a control for both decreased platelet adherence and platelet aggregation. Two independent experiments with two different donors were performed for each panel. Platelet adherence was analyzed by one-way ANOVA (A) and was analyzed by two-way ANOVA (B), both corrected for multiple comparisons by the method of Dunnett. *, *P* < 0.05. Error bars represent the standard deviation.

As *in vitro* experiments demonstrated that T4P could mediate twitching motility on plates, and T4P have been shown to mediate a variety of virulence interactions in other bacteria (25, 28, 29, 35–37), we examined whether S. sanguinis SK36 T4P play a role in platelet adherence and aggregation, which are integral steps in bacterial endocarditis. We recently used these types of experiments to identify S. sanguinis genes important for platelet interactions and vegetation formation in heart infections (38). For these experiments, we compared SK36 as the wild-type parent strain and the isogenic Δ*pilF* mutant that have not been passaged on blood agar. We tested the ability of the SK36, SK36 Δ*pilF*, and SK36 Δ*pbrA* (a nonadhering/nonaggregating control strain) strains (38) to bind to human platelets. To account for differences between platelet donors, we calculated the mutant adherence level relative to the WT. As shown in Fig. 4A, both the WT and Δ*pilF* mutant strain had similarly high levels of platelet adherence while the Δ*pbrA* mutant had significantly reduced levels (∼20%) of adherence to human platelets, as expected. In the platelet aggregation assay, similar results were observed. As seen in Fig. 4B, the WT and Δ*pilF* mutant strain exhibited comparable levels of platelet aggregation while the Δ*pbrA* mutant had virtually no detectable aggregation of human platelets under these conditions.

### Type IV pili mediate internalization, but not adherence, of human aortic endothelial cells.

**FIG 5:**
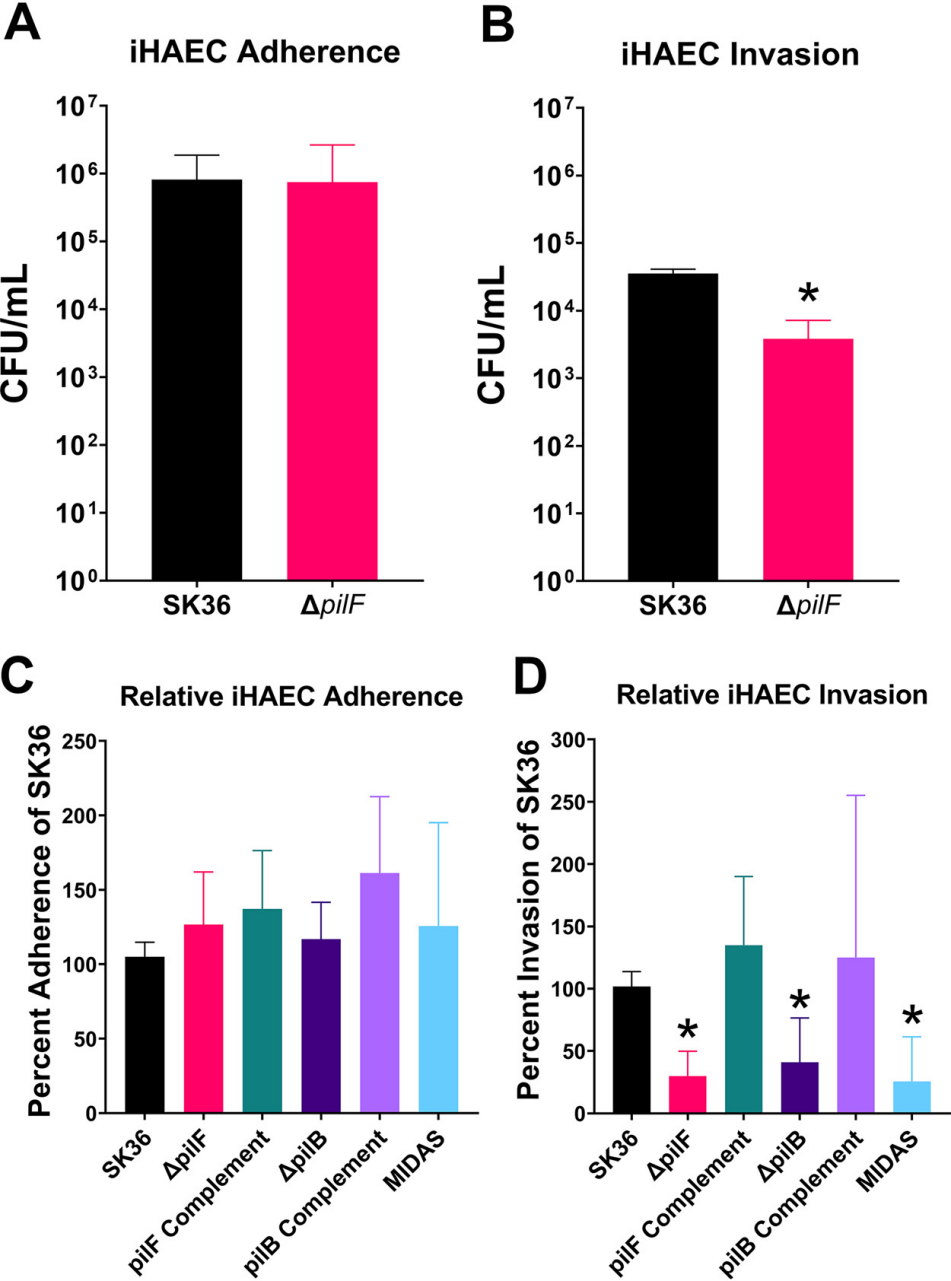
T4P are required for invasion of iHAECs but are dispensable for adherence to iHAECs. (A) Adherence of S. sanguinis SK36 or Δ*pilF* mutant to iHAECs. Bacteria were mixed in a ratio of 50:1 (bacteria/iHAECs) and allowed to bind to the iHAEC surface for 3 h. Wells were washed to remove planktonic bacteria before lysing and plating dilutions of cell-associated bacteria to quantitate adherence levels. (B) Invasion of S. sanguinis SK36 or Δ*pilF* mutant into iHAECs. Bacteria were mixed in a ratio of 50:1 (bacteria/iHAECs) and allowed to interact with the cells. At the end of 3 h, wells were washed three times, and the M200 medium was replaced with fresh M200 medium supplemented with 100 μg/ml of gentamicin for 1 h. Subsequently, the cells were washed three times, lysed, and dilutions were plated to quantitate intracellular CFU. (C) Representative invasion of iHAEC cells by complementation strains and the Δ*pilB*/MIDAS mutants relative to WT invasion, performed as in panel B. Percent adherence and invasion were determined by contrasting the CFU observed following the assay to the CFU observed from plating the inoculum. At least two independent experiments were performed for panel A, and three independent experiments were performed for panel B. At least five independent experiments were performed for panels C and D. Statistical analysis was performed using two-tailed, unpaired *t* tests for panels A and B. Panels C and D were analyzed via one-way Brown-Forsythe and Welch ANOVA corrected for multiple comparisons by the method of Dunnett’s T3 test. *, *P* < 0.05. Variance of the WT was determined by normalizing within-experiment replicates to one relative value for each independent experiment. Error bars represent the 95% confidence interval.

As no phenotype was associated with the Δ*pilF* mutant in platelet adherence or platelet aggregation assays, we examined the interactions of the Δ*pilF* strain with immortalized human aortic endothelial cells (iHAECs) (Fig. 5) (39). Although the uninjured endothelium is an unlikely site of primary attachment and colonization, as evidenced by the difficulty of inducing IE in healthy animals (40–42), it has been hypothesized that later interactions contribute to IE pathogenesis by providing a protected intracellular niche for the organisms during vegetation formation (43). Employing an adherence assay, we observed no difference in adherence of WT SK36 and the Δ*pilF* mutant following incubation with iHAECs for 3 h (Fig. 5A). However, we did observe a significant decrease in invasion recovered from iHAECs of the Δ*pilF* mutant compared to that of WT SK36 as measured in a standard gentamicin invasion assay (Fig. 5B). A chromosomal *pilF* complement exhibited a phenotype statistically similar to the WT, indicating that an intact *pilF* operon is necessary for this phenotype. This result suggests that T4P contribute to entry into endothelial cells, which may have important implications in the pathogenesis of infective endocarditis. Growth control experiments revealed neither significant bacterial growth in tissue culture medium over the time course of the experiment nor any difference in gentamicin susceptibility between mutant strains and WT (data not shown).

While this manuscript was in preparation, the minor pilin PilB was described to contain a von Willebrand factor A-like domain (vWA) (44). This domain is known to mediate adherence to a variety of ligands including eukaryotic cells and extracellular matrix molecules (ECM) (45). Further, the vWA domain contains a metal ion-dependent adhesion site (MIDAS) domain that has been shown to be critical for virulence in other organisms (46). Therefore, we also created both an S. sanguinis Δ*pilB* mutant and an S. sanguinis MIDAS domain mutant in the *pilB* gene to test in our invasion assay. The MIDAS domain mutant contains three amino acids substitutions in the DXSXS motif that directly bind the metal ion necessary for adhesion of other molecules (45, 47). Similar to the Δ*pilF* mutant, we observed a significant decrease in bacterial recovery following invasion of iHAECs in both the Δ*pilB* and MIDAS mutants (Fig. 5D) while no difference was observed in adhesion of iHAECs (Fig. 5C). Importantly, complementation of the Δ*pilB* mutant restored the invasiveness of the strain for iHAEC cells to WT levels. To determine whether the *pilB* mutation impacted downstream transcription, we performed reverse transcriptase PCR (RT-PCR) on the *pilC* gene, which is immediately downstream from *pilB*. We observed similar levels of *pilC* transcript between the Δ*pilB* mutant and WT (data not shown). We also confirmed that the Δ*pilB* and MIDAS mutations did not cause a general disruption of T4P production, as we could still detect the structural PilE subunits by Western blotting in these mutants (Fig. 2D), indicating that the simplest explanation for our data is that the PilB subunit is the T4P adhesin and that the MIDAS domain is the region of the PilB subunit mediating adherence.

### Platelet-dependent biofilm formation requires type IV pili.

**FIG 6:**
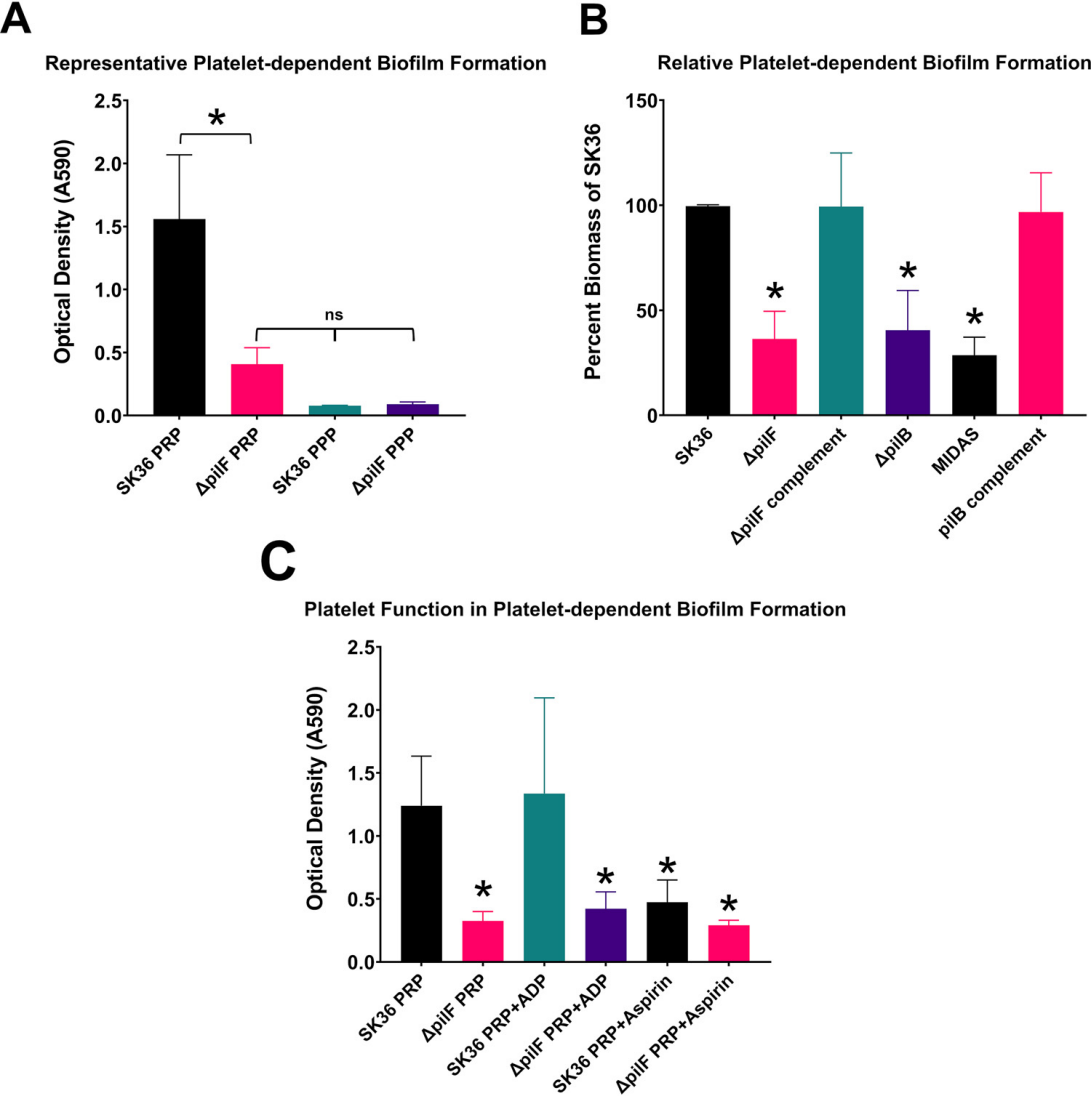
T4P contribute to platelet-dependent biofilm formation and function independently of platelet aggregation. Overnight bacterial cultures were diluted into a 1:1 mixture of TH/PRP and grown for 24 h in an anaerobic chamber at 37°C. Total biomass representing both bacterial and platelet factors were washed, heat fixed, and stained with crystal violet. (A) Representative biomass development in both PRP and PPP from two independent experiments. (B) Relative platelet-dependent biofilm formation in PRP. Values are from seven independent experiments. (C) Effects of ADP and aspirin on platelet-dependent biofilm formation. At least three independent experiments were performed. Statistical analysis in panel A was performed using one-way ANOVA corrected for multiple comparisons by the method of Sidak. Data in panels B and C were analyzed via one-way ANOVA corrected for multiple comparisons by the method of Dunnett. *, *P* < 0.05. Error bars represent the 95% confidence interval.

T4P are known to contribute to biofilm formation in various bacterial species (26, 48), but the application of *in vitro* microtiter biofilm assays to IE virulence is not always clear (49, 50). We therefore sought to determine whether T4P of S. sanguinis could contribute to biofilm development in an environment that also includes human platelets. Platelet-dependent biofilms resemble vegetations developed during IE and have been successfully reproduced *in vitro* (51–53). WT SK36 incubated with platelet-rich plasma (PRP) from healthy human donors consistently produced biofilms of significant biomass compared to those of the Δ*pilF* mutant or platelet-poor plasma (PPP) control (Fig. 6). Due to variability observed among random platelet donors, the data from these experiments is presented both as a representative experiment (Fig. 6A) and as standardized data in which SK36 platelet-dependent biofilm formation was arbitrarily set to 100% so that each experiment could be compared to each other, despite blood donor variations (Fig. 6B). These experiments reproducibly demonstrated that the Δ*pilF*, Δ*pilB*, and MIDAS domain mutations induced ∼35 to 40% of platelet-dependent biofilm formation compared to WT (100%), and complementation of each of the mutations restored the phenotype to WT levels. These data indicate that both an intact *pilF* operon and a functional *pilB* gene with an intact MIDAS domain are necessary to induce platelet-dependent biofilm or aggregation.

To explore this phenotype in more depth, we performed experiments with ADP, a platelet aggregation agonist, and aspirin, a platelet function inhibitor of aggregation. The WT strain assayed in the presence of ADP generally produced biofilms of greater biomass than those without, but this was not determined to statistical significance. In contrast, a Δ*pilF* mutant grown with ADP-stimulated platelets did not produce biofilms of greater biomass (Fig. 6C). Conversely, WT strains grown in the presence of aspirin produced biofilms of significantly less biomass compared to those grown in its absence, while the Δ*pilF* strain produced biofilms of similar low biomass regardless of the presence of aspirin (Fig. 6C).

### T4P pili are critical for pathogenesis in experimental infective endocarditis.

**FIG 7:**
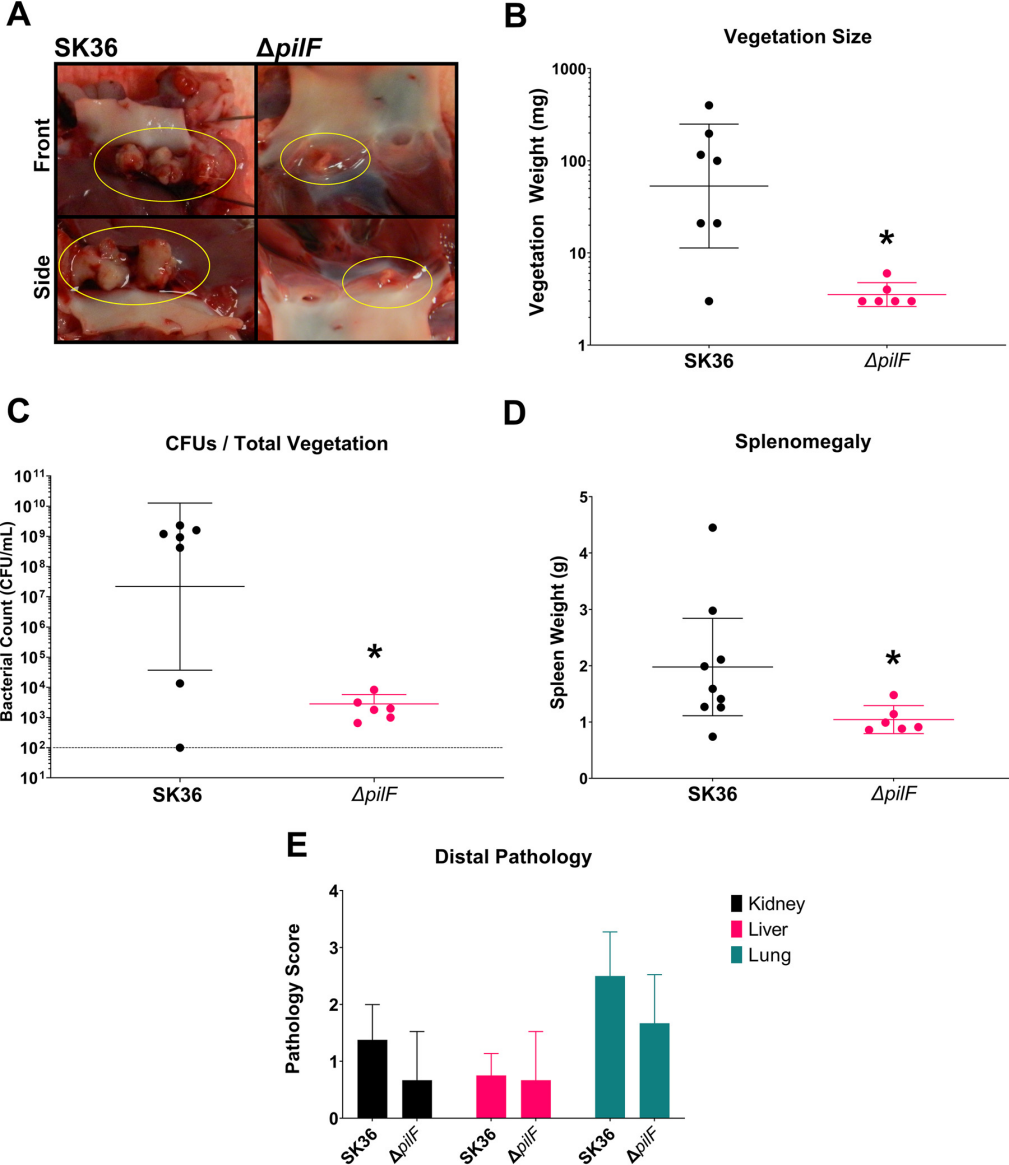
Disease pathology of infective endocarditis is significantly attenuated in rabbits infected with the Δ*pilF* mutant. (A) Representative images of cardiac vegetations from WT (left) and Δ*pilF* mutant (right) rabbits with lesions encircled. (B) Vegetation sizes from experimental rabbits. All except one rabbit infected with WT developed vegetations greater than 20 mg; no vegetations larger than 10 mg were observed in rabbits infected with the Δ*pilF* mutant, with the average being 3.5 mg. (C) CFU recovered from cardiac lesions or the valve surface are significantly different between WT and mutant strains. (D) Rabbits infected with the mutant strain experienced significantly less splenomegaly as measured by spleen weight. Means for vegetation size and CFU are geometric. Statistical analysis was performed using Mann-Whitney. *, *P* < 0.05. Error bars represent the 95% confidence interval. Two additional data points for the WT strain are present due to the hearts of two rabbits being sent for histology; vegetations from these rabbits were thus not able to be quantified during necropsy. (E) Pathology of distal organs is not significantly different between WT and the Δ*pilF* mutant. Gross pathology of the kidney, liver, and lungs from infected rabbits scored by a previously described scale (38). Organs were scored by three blinded investigators. The SK36 Δ*pilF* mutant did not exhibit distinct organ pathology compared to WT. Kidneys and liver exhibited moderate pathology on average, characterized by rare, small lesions evident on the surface. By contrast, lungs generally exhibited more severe pathology characterized by multifocal hemorrhaging and necrosis evident on one or both lobes. Statistical analysis was performed using unpaired, two-tailed *t* tests. Error bars represent the 95% confidence interval.

To determine whether the *in vitro* phenotypes that we observed for the T4P would translate to *in vivo* virulence phenotypes, we performed infection experiments with the nonpiliated Δ*pilF* mutant in a New Zealand White rabbit model of left-sided native valve IE (54). We have previously demonstrated the efficacy of this model to investigate IE caused by S. sanguinis and its resemblance to human disease (38). Rabbits infected with the Δ*pilF* mutant were observed to develop cardiac vegetations of significantly smaller size (median 3 mg/vegetation) than those of the WT (median 100 mg/vegetation) (Fig. 7A and B). Lesions present in mutant-infected rabbits largely resembled thrombi expected from catheter-induced aortic damage (i.e., small platelet-fibrin aggregates at sites of endothelial injury; see Werdan et al. (43) for a discussion of native-valve colonization and IE progression) rather than the expansive pathological lesions caused by WT SK36 colonization, suggesting that the S. sanguinis Δ*pilF* mutant is unable to induce vegetation development in the rabbit model of endocarditis (Fig. 7A). Valve surfaces and lesions in the mutant-infected rabbits also contained several logs fewer bacteria than the WT, indicating limited bacterial presence (Fig. 7C). Importantly, *in vitro* growth of the Δ*pilF* mutant in rabbit serum was not different from that of the WT (see Fig. S3 in the supplemental material), indicating that these differences are not due to increased sensitivity to serum or altered *in vivo* growth kinetics. These results demonstrate that T4P are essential for the S. sanguinis endocarditis strain SK36 to develop large vegetations during disease. Additionally, spleens from these animals exhibited significantly reduced size (Fig. 7D), consistent with a reduced bacterial burden. Finally, we examined gross pathology in the kidneys, liver, and lungs. There was little detectable pathology observed in distal organs, and there was no significant difference in pathology scores between rabbits infected with WT SK36 and the Δ*pilF* mutant (Fig. 7E).

## DISCUSSION

Type IV pili are versatile bacterial structures that function in a wide range of microbial processes. The roles played by T4P in Gram-negative physiology and virulence have been explored in detail, but the functions of T4P in Gram-positive organisms are only beginning to be appreciated (23, 26, 55). Indeed, even though twitching motility in S. sanguinis was first observed in 1975 (20), it took over 40 years for this phenomenon to be genetically characterized and demonstrated to be mediated by T4P (30). Although substantial progress has been made in defining the molecular mechanisms and assembly of this system in S. sanguinis (31, 32, 44), the role of T4P in S. sanguinis endocarditis has not been described previously.

Our work here identifies T4P as another significant S. sanguinis endocarditis virulence factor, as we have demonstrated that T4P of S. sanguinis are critical for the pathogenesis (development of cardiac vegetations) observed in a native valve rabbit model of IE (Fig. 7). Furthermore, *in vitro* experiments identify possible mechanisms by which T4P may contribute to aortic heart valve infections. We found that a nonpiliated Δ*pilF* mutant of S. sanguinis SK36 adhered as well as the WT to iHAECs (Fig. 5A) but exhibited reduced intracellular infection of the same cells (Fig. 5B), which we attribute to a reduction in invasion. It is possible that greater numbers of internalized bacteria are present in the Δ*pilF* mutant due to differences in killing, but we consider this possibility remote due to the short incubation time during gentamicin treatment and previous observations on internalized oral streptococci (56). Invasion of endothelial cells is hypothesized to promote inflammation and persistence during disease (43), suggesting S. sanguinis—a typically extracellular bacteria—could take advantage of an intracellular environment to evade host immunity, promote inflammation, and prolong colonization in the circulating system. The direct contribution of these mechanisms to disease progression will be assessed in future research efforts.

Platelet-dependent biofilm formation (a model for vegetation development) was significantly reduced in a Δ*pilF* mutant compared to WT (Fig. 6A and B), indicating a potential role for T4P in vegetation development. Our results also indicate that T4P do not play a critical role in the early interactions of platelet binding and aggregation in endocarditis but rather at later stages, following aggregation. ADP-induced platelet activation fails to rescue biofilm development in the Δ*pilF* mutant (Fig. 6C), suggesting a specific role for T4P in modulating the development of platelet-dependent biofilms since platelets are induced to aggregate under this condition. Furthermore, the ability of aspirin to suppress platelet-dependent biofilm formation (Fig. 6C) demonstrates the requirement of platelet aggregation in the T4P-mediated events of biofilm formation.

Recently published work has identified the presence of a vWA domain in the minor pilin subunit, PilB (44). The normal physiological role of von Willebrand factor protein is in hemostasis, where it initiates binding to platelets via the type A1 domain and GPIβ platelet receptor under high shear stress (57, 58). This interaction is known to be a critical component of IE pathogenesis (59–63), suggesting that this binding activity may be the contribution of T4P to vegetation development and disease. Interestingly, previous research has also established that S. sanguinis SrpA interaction with platelets is similarly mediated through GPIβ via sialic acid residues (64), but this interaction occurs most efficiently under low-shear conditions (65). Although we did not observe a contribution for T4P in binding to platelets or aortic endothelial cells under static conditions (Fig. 5 and 6), we hypothesize that PilB could mediate attachment to a developing thrombus on damaged endothelium under high-shear flow conditions. Similarly, binding to GPIβ via the MIDAS domain (47) could provide a scaffold to develop the vegetation following attachment and colonization. Indeed, we observed a loss of platelet-dependent biofilm formation in both the Δ*pilB* and MIDAS mutants (Fig. 6B), indicating that this domain of PilB mediates interactions with platelets. In addition, this domain also appears to mediate interactions that facilitate bacterial invasion of endothelial cells (Fig. 5D).

While this manuscript was being revised, another group published a structural and functional characterization of the PilB protein and associated MIDAS domain in the S. sanguinis strain 2908 (66). Consistent with our observations, they report functional interaction between the MIDAS domain and epithelial (Chinese hamster ovary) cells, as well as binding to the host matrix molecules fibrinogen and fibronectin (66). However, in contrast to our observations, we did not observe any clear impairment of twitching motility in either the *pilB* or MIDAS mutants of strain SK36. Further, we were able to successfully delete the *pilB* gene in SK36 without loss of filament production, in contrast to previous results in strain 2908 from the same group (30). Possible explanations for these apparent discrepancies include genetic differences between the strains, differences in assay conditions, and mutagenesis strategies. Despite these contrasts, the *pilB* gene, and more specifically the MIDAS domain, of SK36 similarly possesses functional properties related to interaction with human aortic endothelial cells and platelets. Whether these *in vitro* functions translate to *in vivo* virulence will require additional investigations.

In studies aimed at understanding T4P production and function, we found that S. sanguinis SK36 both produces T4P (Fig. 2) and is capable of twitching motility following passage on blood agar plates (Fig. 3). The functional contribution of S. sanguinis SK36 T4P to disease, in a strain that does not easily express T4P motility, suggests that macroscopic surface motility is a T4P activity that does not necessarily correlate with the virulence properties of T4P. Consistent with this hypothesis, SK36 isolated from cardiac vegetations following experimental IE do not exhibit surface motility comparable to strains passaged on blood agar (compare Fig. S4 in the supplemental material and the rightmost panels of Fig. 3). Interestingly, although our experiments indicate that iron does not modulate twitching motility under these conditions (Fig. 3), another group recently observed reduced expression of *pilF*, *pilT*, and other genes in the T4P operon upon depletion of manganese (67). Along with prior work demonstrating the importance of manganese in the virulence of S. sanguinis (68), these results suggest that the acquisition of manganese and/or other ions by SsaB could contribute to the regulation of T4P.

The S. sanguinis virulence strategy for IE is dependent, like many oral streptococci, on its broad multicomponent adhesive properties for human platelets (11, 12, 69) and extracellular matrix molecules (70). Some early evidence suggested a role for conventional biofilm processes, such as EPS production (13, 71), in disease, but a direct correlation between *in vitro* biofilm formation and pathogenesis has not been corroborated in subsequent work (72). More recent work has added significant detail to this model, and multiple investigations have also defined integral redox processes as critical to IE pathogenesis (68, 73, 74). In addition, several virulence factors mediating interactions with platelets and expected to function in the earlier stages of disease (38, 64, 75) or as being immunosuppressive (76) have been identified and characterized in greater detail. Based on the work presented here, we hypothesize another layer to this model whereby factors such as T4P contribute directly to vegetation development and maturation at later stages of disease. Mechanistic studies are under way in our laboratory to further evaluate this prospect.

Taken together, our data demonstrate that T4P of S. sanguinis are a critical virulence factor in native valve infective endocarditis and contribute to pathogenesis through the development of cardiac vegetation. Furthermore, our data indicate that the MIDAS domain of PilB is responsible for mediating this interaction with human platelets. It is also possible that through invasion of aortic endothelial cells and intracellular persistence T4P contribute to persistent inflammation and recruitment of host factors; however, the contribution of intracellular persistence to IE pathogenesis still needs to be tested experimentally. We have also demonstrated that T4P mediate twitching motility in S. sanguinis SK36, which contrasts with previous reports (30, 31). Our results indicate that virulence and disease phenotypes mediated by T4P in S. sanguinis exist independently from twitching motility observed on agar plates.

## MATERIALS AND METHODS

### Bacterial strains and growth conditions.

S. sanguinis strain SK36, a human dental plaque isolate (77), was generously provided by Mark Herzberg (School of Dentistry, University of Minnesota). Routine culturing was performed at 37°C under stationary conditions in Todd-Hewitt broth (TH) (Dot Scientific, Burton, MI) or on brain heart infusion (BHI) (Research Products International [RPI], Mt. Prospect, IL) supplemented with 1.5% agar (BD; Becton, Dickinson and Company, East Rutherford, NJ) (referred to here as BHA) in an atmosphere containing 5% CO_2_. Anaerobic experiments were performed in an anaerobic chamber (Coy Laboratory Products) with an atmosphere of 10% H_2_, 5% CO_2_, and 85% N_2_. For a list of strains used in this study, see Table 1.

**TABLE 1:** List of strains used in this study

Strain	Description	Reference or source
SK36	Dental plaque isolate	75
SK36 Δ*pilF*::*erm*	Isogenic *pilF* deletion of SK36	This study
SK36 Δ*pilF*::*erm* × *pilF*	Complement of Δ*pilF*::*erm*	This study
SK36 Δ*pbrA*::*kan*	Isogenic *pbrA* deletion of SK36	38
2908	Throat isolate	20, 30
2908 ΔpilF::*kan*	Isogenic *pilF* deletion of 2908	30
SK36 Δ*pilB*::pheS*-kan	Isogenic *pilB* deletion of SK36	This study
SK36 Δ*pilB*	Isogenic-markerless *pilB* deletion of SK36	This study
SK36 Δ*pilB*::pheS*-kan X pilB	Complement of Δ*pilB*::pheS*-kan	This study
SK36 pilB-MIDAS	Point mutations in PilB D304A, S306A, and S308A	This study
SK36 Δ*ssaB*::*kan*	Isogenic *ssaB* deletion of SK36	38

### Construction of mutant strains.

Primers used for mutagenesis are listed in Table 2. The Δ*pilF* mutant was constructed using previously described methods (30), using an erythromycin resistance gene cassette for gene replacement instead of a spectinomycin cassette. The Δ*pilB* and MIDAS mutants were transformed directly with a counterselectable pheS*-kan system previously described for S. sanguinis (78). Transformants were selected on BHA supplemented with 500 μg/ml of kanamycin and then PCR screened for the presence of the cassette using the listed upstream F1 and downstream R3 primers. The Δ*pilB*::pheS*-kan strain was independently transformed with different SOE PCR products to yield either a clean Δ*pilB* mutant expressing the first 2 and last 10 amino acids or an altered MIDAS domain where residues D304, S306, and S308 were mutated to alanines. The pheS*-kan construct was assembled and mutagenized using previously described methods (79, 80). Complementation of the Δ*pilF* and Δ*pilB* mutants were performed using the same counterselection method in combination with genomic transformation to restore the native gene. Briefly, the erythromycin cassette in the Δ*pilF*::*erm* mutant was first replaced with a pheS*-kan cassette. Confirmed Δ*pilF*::pheS*-kan mutants were then transformed with purified genomic DNA isolated from WT S. sanguinis SK36 and plated on BHA supplemented with 15 μM *p*-chlorophenylalanine. In the case of the Δ*pilB* mutant, the gene directly replaced the initial pheS*-kan cassette and was verified by PCR. We tested both the clean and pheS*-kan Δ*pilB* mutants and observed identical phenotypes (data not shown); therefore, the clean deletion is represented in associated figures. All PCRs were performed using Q5 high-fidelity DNA polymerase (NEB; Ipswich, MA) according to the manufacturer’s instructions. The MIDAS point mutations were confirmed by Sanger sequencing.

**TABLE 2:** List of primers used in this study

Primer	5′–3′ sequence^*a*^
Upstream_PilF.Deletion.F1	CTTTGGTCGATAACATTGCG
Upstream_PilF.Deletion.R1	*CCTATTTTTTGTGAATCG* **GCAATGAAGATTGCCTTCAG**
ErmC_PilF.Deletion.F2	*GAAGGCAATCTTCATTGC* **CGATTCACAAAAAATAGGCACAC**
ErmC_PilF.Deletion.R2	*CACAGACAGGAGAAACAA* **ATGAACGAGAAAAATATAAAACACAGTC**
Downstream_PilF.Deletion.F3	*GACTGTGTTTTATATTTTTCTCGTTCAT* **TTGTTTCTCCTGTCTGTG**
Downstream_PilF.Deletion.R3	AAGCTGAGTACATTGTGC
SK36_PheS.F1	*GCTGGAACTACTGTCTTTCT* **ATGACGAAAACGATTGAAGA**
SK36_PheS.R1	*AGCCATTGATAATATCTCCT* **CTACTTAAACTGCTGAGAAAAAC**
KanR.F2	*GCAGAAACCTTGTTCATCAA* **CTAAAACAATTCATCCAGTAAAA**
KanR.R2	*AAGTAGAGGAGATATTATCA* **ATGGCTAAAATGAGAATATCAC**
pDL278_Linearization.F3	*TACTGGATGAATTGTTTTAG* **TTGATGAACAAGGTTTCTGC**
pDL278_Linearization.R3	*TCTTCAATCGTTTTCGTCAT* **AGAAAGACAGTAGTTCCAGC**
PheS-Kan_SDM(A316G).F4	*GAGAAATACTCTGGATTTGGT* **TTTGGTCTCGGTCAAGAGCG**
PheS-Kan_SDM(A316G).R4	ACC**AAATCCAGAGTATTTCTCTGCATCAATGC**
Upstream_PilF.PheS*-Kan.F1	CTTTGGTCGATAACATTGCG
Upstream_PilF.PheS*-Kan.R1	*GAATTGTTTTAGTTGATG* **GCAATGAAGATTGCCTTC**
PilF::PheS*-Kan.F2	*CACAGACAGGAGAAACAA* **ATCCCATTATGCTTTGGCAG**
PilF::PheS*-Kan.R2	*GAAGGCAATCTTCATTGC* **CATCAACTAAAACAATTC**
Downstream_PilF.PheS*-Kan.F3	*CTGCCAAAGCATAATGGGAT* **TTGTTTCTCCTGTCTGTG**
Downstream_PilF.PheS*-Kan.R3	AAGCTGAGTACATTGTGC
Upstream_PilB.Deletion.F1	AACTGAATTTTGGAGTCGTTAAGAG
Downstream_PilB.Deletion.R3	CTTGGTTGAGTTGATTGTCGTAAT
Downstream_PilB.PheS*-Kan.R1	*TACTGGATGAATTGTTTTAG* **CAAGATCTTTGGTTTGTGTC**
	
Upstream_PilB.PheS*-Kan.F2	*GACACAAACCAAAGATCTTG* **CTAAAACAATTCATCCAGTA**
Downstream_PilB.PheS*-Kan.R2	*TTAAATAGGAAAGAATGATG* **ATCCCATTATGCTTTGGCAG**
Upstream_PilB.PheS*-Kan.F3	*CTGCCAAAGCATAATGGGAT* **CATCATTCTTTCCTATTTAA**
Downstream_PilB.CD.R1	*TTAAATAGGAAAGAATGATG* **CAAGATCTTTGGTTTGTGTC**
Upstream_PilB.CD.F2	*GACACAAACCAAAGATCTTG* **CATCATTCTTTCCTATTTAA**
Downstream_PilB.MIDAS.R1	*CAGCTGTTTCCTTCGTTTTT* GCT *ACA* GCA *GGT* GCC **ATGGCTTATGGATTAAGGAATG**
Upstream_PilB.MIDAS.F2	*CATTCCTTAATCCATAAGCCAT* GGC *ACC* TGC *TGT* AGC **AAAAACGAAGGAAACAGCTG**

a Segments highlighted in bold indicate the target sequence, while segments highlighted in italics indicate homologous recombination sequences. Underlined segments are the codon sequences used for site-directed mutagenesis.

### Twitching motility.

The ability of S. sanguinis strains to exhibit twitching motility on agar plates was assessed by cultivation on 80% strength Levinthal’s medium base (HiMedia Laboratories, Mumbai, India) supplemented with defibrinated sheep blood (Colorado Serum Company, Denver, CO) to a final concentration of 5%. Plates were poured and allowed to dry for 1 h on the benchtop and <5 min in a biological safety hood prior to inoculation. Strains grown overnight on BHA were lightly streaked onto blood agar using sterile toothpicks. Plates were incubated in a sealed candle jar containing sterile water at the bottom for 4 to 5 days at 37°C. Successive passaging was performed as follows. Bacteria at the edges of the prior colony streaks were gently scraped with a sterile toothpick and restreaked onto fresh plates prepared as described above. In experiments using iron or chelation supplements, colonies were restreaked onto plates containing the same supplement as the original plate. Colony isolation experiments were performed using conventional BHA, and individual colonies were propagated via broth culture in TH.

### Pilus shearing and immunoblotting.

Shearing of surface pili was performed as described previously with minor modifications (30). Briefly, overnight cultures were inoculated 1:200 into 100 ml TH and incubated for the indicated period of time at 37°C in a CO_2_ incubator. Cell concentrations were normalized to an OD_600_ of 1.0, and then identical volumes were pelleted by centrifugation at 6,000 relative centrifugal force (rcf) for 10 min at 4°C. Cell pellets were resuspended in a Tris-NaCl buffer and transferred to 2-ml tubes for shearing. Pili shearing was performed by mixing on a vortex at maximum speed three times for 1 min each time. Cell debris was pelleted by centrifugation at 6,000 rcf for 5 min at 4°C, and the supernatant transferred to ultracentrifuge tubes. Supernatants were then ultracentrifuged at 100,000 rcf at 4°C for 1 h, the supernatant was aspirated, and the protein pellet resuspended in 20 μl Tris-NaCl buffer for analysis by Coomassie stain or immunoblotting. Colloidal Coomassie was prepared, and the staining performed as described for the method of Kang by Dyballa and Metzger (81).

Rabbit IgG polyclonal antibodies toward the PilE subunits were individually generated by ProSci against peptide antigens in two different animals. Peptides were synthesized as follows: PilE1 (CDTLATDGKDKHAA), PilE2 (CKGTLADTLAKDGANNGPA), and PilE3 (CGELSKTLAADNGKIAHE). Immune responses were validated via enzyme-linked immunosorbent assay (ELISA), and pooled serum from the first and final bleeds was used at a 1:200 dilution for immunoblotting. Serum antibodies were detected using IRDye 680LT goat anti-rabbit (1:10,000; Li-Cor) and imaged on an Odyssey CLx (Li-Cor).

### Platelet adherence and aggregation.

Platelet adherence and aggregation were performed essentially as described previously (38). Platelets were obtained using fresh whole blood collected in BD Vacutainer tubes (3.2% sodium citrate) from human donors. Binding to human platelets was determined by resuspending overnight cultures of WT S. sanguinis and the Δ*pilF* mutant in phosphate-buffered saline (PBS) to approximately 2.5 × 10^8^ CFU/ml. The 100-μl aliquots (∼2.5 × 10^7^ CFU/well) were distributed to wells of a 96-well microtiter plate incubated for 2 h at 37°C to permit bacterial adherence. Inoculated wells were then washed and blocked with 1% bovine serum albumin (BSA) in PBS for 1 h at 37°C. Wells were washed with Tyrode’s buffer before addition of approximately 2.5 × 10^7^ platelets, at which point plates were incubated for 30 min at 37°C. Nonadherent platelets were removed by washing three times with Tyrode’s buffer, and bacteria-bound platelets were lysed by the addition of 100 μl lysis buffer containing 10 mM *p-*nitrophenylphosphate (pNPP). Plates were incubated for 30 min at 37°C and reactions stopped by the addition of 100 μl 1 M NaOH. Production of yellow nitrophenol was quantified at an absorbance of 410 nm using a plate reader.

Aggregation was quantified via a modified light transmission aggregometry assay. Platelets in PRP were enumerated via 100-fold dilution in 1% ammonium oxalate and adjusted to a concentration of 3 × 10^8^ to 4 × 10^8^/ml using autologous PPP. Concurrently, overnight cultures of S. sanguinis were pelleted and the supernatant removed by aspiration. Cell pellets were resuspended in filter-sterilized isotonic glucose and normalized to an OD_600_ of 10.0 in the same solution. S. sanguinis suspensions were distributed in 10-μl aliquots to a 96-well microtiter plate in triplicate. Solutions of isotonic glucose alone or containing 0.5 μg/ml collagen type I (CHRONO-LOG, Havertown, PA) were similarly distributed as negative and positive controls, respectively. Once the plate reader reached 37°C, 90 μl of either PRP or PPP was rapidly added to one concomitant half of each experimental and control condition. Platelet aggregation was monitored by reading the OD_595_ immediately after addition of PRP or PPP and every 150 s thereafter for 40 min.

### Endothelial cell adherence and invasion.

Adherence and invasion assays of immortalized aortic endothelial cells (iHAEC) (39) were performed largely as previously described (82). HAECs were cultured in Medium 200 containing 10% low-serum growth supplement (LSGS) (Thermo Fisher, Waltham, MA). The 24-well tissue culture plates (CoStar 3596; Corning Incorporated, Corning, NY) were seeded in triplicate with approximately 5 × 10^4^ cells and incubated for 48 h at 37°C in a 5% CO_2_ environment prior to bacterial inoculation. Overnight cultures of S. sanguinis were washed once in PBS and an equivalent volume added to each well at a multiplicity of infection (MOI) of 50; cells were incubated for an additional 3 h as above. Parallel plates were set up to separately evaluate bacterial adherence and invasion. To determine bacterial adherence, infected cells were washed three times to remove nonadherent bacteria and then lysed by incubation in 200 μl of 0.025% trypsin-EDTA for 5 min at room temperature (RT), followed by the addition of 800 μl PBS containing 1% Tween 20 for 20 min at 37°C. Lysates were mixed vigorously by pipetting and dilutions plated on BHA to enumerate CFU. Bacterial invasion was determined by washing cells as above and then incubating for 1 additional hour in medium containing 100 μg/ml gentamicin. Following gentamicin treatment to kill extracellular bacteria, cells were washed to remove gentamicin and lysed, and portions of the lysate were plated to quantitate intracellular bacteria.

### Platelet-dependent biofilm formation.

The ability of T4P to induce a platelet-dependent biofilm representing a thrombus was evaluated using a previously reported method (51) with some modifications. PRP was generated from citrated whole blood by centrifugation at 200 rcf for 10 min, and autologous PPP was generated by centrifugation of the remaining blood components at 2,000 rcf for 20 min. Bacterial strains grown overnight in TH were washed once in PBS and normalized to an OD_600_ of 1.0, and 20 μl was distributed to wells of a 96-well plate in triplicate. Prior to inoculation, PRP was mixed 1:1 with TH and 180 μl distributed to each condition of the 96-well plate before plates were parafilmed to minimize evaporation and incubated for 24 h at 37°C in an anaerobic chamber. In experiments with ADP or aspirin, ADP was added to a final concentration of 24 μM and aspirin to a final concentration of 50 μg/ml. After 24 h, plates were decanted and washed three times by submersion in distilled water, heat fixed for 45 min at 60°C, and stained with 0.1% crystal violet for 15 min. Crystal violet was decanted, and plates were washed by submersion in distilled water to remove excess crystal violet. Plates were then inverted and allowed to dry for 5 min before solubilizing the dye by addition of 150 μl 40 mM HCl in 95% ethanol for 30 min on the benchtop. Solubilized crystal violet was transferred to unused wells and the OD_590_ read using a microtiter plate reader to quantitate platelet biofilm for each well (Tecan; Infinite M200 PRO).

### Serum growth experiments.

Growth experiments using rabbit serum were performed largely as previously described (68), with some modifications. Briefly, strains were grown overnight in 80% TH/20% rabbit serum (Corning Life Science, Corning, NY) and then adjusted to an OD_600_ of 1.0. The strains were then diluted 1:10,000 into 100% rabbit serum and aliquoted in triplicate into a 96-well plate. Each sample was covered with sterile heavy mineral oil, and the OD_600_ was read using a microtiter plate reader every 20 min for 15 h.

### Experimental infective endocarditis.

Pathogenesis of the nonpiliated SK36 *pilF*::*erm* strain was tested using an outbred rabbit model of aortic injury as described previously (38, 54). Briefly, rabbits were anesthetized and the carotid artery isolated, occluded at the distal end with sutures, and incised to permit introduction of a sterile catheter. The inserted catheter was extended down the carotid until contact with the aorta was achieved and then tied in place to mechanically abrade the valve for 2 h. The catheter was then removed, and the carotid artery was tied off with sutures. Bacterial strains were prepared from overnight cultures by washing and resuspension in hospital-grade saline to approximately 1 × 10^8^ CFU/ml, and 2 ml of this solution was used to induce bacteremia via intravenous injection through the marginal ear vein. Rabbits were monitored for 7 days postinfection. Surviving animals were euthanized at day seven and necropsied for the development of cardiac vegetations and disseminated organ pathology.

### Ethical statement.

All animal experiments were performed in accordance with the protocols of the Institutional Animal Care and Use Committee of the University of Iowa (protocol number 1106138). The University of Iowa is a registered research facility with the U.S. Department of Agriculture (USDA) and complies with the Animal Welfare Act and Regulations (AWA/AWR). The facility holds an Animal Welfare Assurance through the Office of Laboratory Animal Welfare (OLAW) and complies with PHS Policy. Additionally, the facility is accredited through the Association for Assessment and Accreditation of Laboratory Animal Care International (AAALACi) and is committed to comply with the Guide for the Care and Use of Laboratory Animals.

Human platelets were acquired from the University of Iowa DeGowin Blood Center as platelet-rich plasma (PRP). We have received approval from the University of Iowa Institutional Review Board to use human blood components under protocol number 201902770. All samples used for this work were anonymized. The patients/participants provided their written informed consent to participate in this study. All participants were over 16 years of age.

### Statistical analysis.

Statistical analysis of data was performed using GraphPad Prism 8 (GraphPad Software, San Diego, CA, United States). Significance was determined by one-way analysis of variance (ANOVA), two-way ANOVA, Brown-Forsythe and Welch ANOVA, Mann-Whitney U test, or unpaired *t* test as indicated in the figure legends. Values indicate the mean ± 95% confidence interval (CI) or standard deviation (SD), as noted for each figure. *P *< 0.05 was considered significant for statistical hypothesis testing.

## References

[1] Holland TL, Baddour LM, Bayer AS, Hoen B, Miro JM, Fowler VGJ. 2016. Infective endocarditis. Nat Rev Dis Primers 2:16059. doi:10.1038/nrdp.2016.59. 27582414 PMC5240923

[2] Murdoch DR, Corey GR, Hoen B, Miró JM, Fowler VGJ, Bayer AS, Karchmer AW, Olaison L, Pappas PA, Moreillon P, Chambers ST, Chu VH, Falcó V, Holland DJ, Jones P, Klein JL, Raymond NJ, Read KM, Tripodi MF, Utili R, Wang A, Woods CW, Cabell CH. 2009. Clinical presentation, etiology, and outcome of infective endocarditis in the 21st century: the International Collaboration on Endocarditis-Prospective Cohort Study. Arch Intern Med 169:463–473. doi:10.1001/archinternmed.2008.603. 19273776 PMC3625651

[3] Njuguna B, Gardner A, Karwa R, Delahaye F. 2017. Infective endocarditis in low- and middle-income countries. Cardiol Clin 35:153–163. doi:10.1016/j.ccl.2016.08.011. 27886786

[4] Baddour LM, Wilson WR, Bayer AS, Fowler VG, Tleyjeh IM, Rybak MJ, Barsic B, Lockhart PB, Gewitz MH, Levison ME, Bolger AF, Steckelberg JM, Baltimore RS, Fink AM, O'Gara P, Taubert KA, American Heart Association Committee on Rheumatic Fever, Endocarditis, and Kawasaki Disease of the Council on Cardiovascular Disease in the Young, Council on Clinical Cardiology, Council on Cardiovascular Surgery and Anesthesia, and Stroke Council. 2015. Infective endocarditis in adults: diagnosis, antimicrobial therapy, and management of complications: a scientific statement for healthcare professionals from the American Heart Association. Circulation 132:1435–1486. doi:10.1161/CIR.0000000000000296. 26373316

[5] Habib G. 2006. Management of infective endocarditis. Heart 92:124–130. doi:10.1136/hrt.2005.063719. 16365367 PMC1861013

[6] Paterick TE, Paterick TJ, Nishimura RA, Steckelberg JM. 2007. Complexity and subtlety of infective endocarditis. Mayo Clin Proc 82:615–621. doi:10.4065/82.5.615. 17493427

[7] Thuny F, Grisoli D, Collart F, Habib G, Raoult D. 2012. Management of infective endocarditis: challenges and perspectives. Lancet 379:965–975. doi:10.1016/S0140-6736(11)60755-1. 22317840

[8] Cahill TJ, Baddour LM, Habib G, Hoen B, Salaun E, Pettersson GB, Schäfers HJ, Prendergast BD. 2017. Challenges in infective endocarditis. J Am Coll Cardiol 69:325–344. doi:10.1016/j.jacc.2016.10.066. 28104075

[9] Yew HS, Murdoch DR. 2012. Global trends in infective endocarditis epidemiology. Curr Infect Dis Rep 14:367–372. doi:10.1007/s11908-012-0265-5. 22592632

[10] Herzberg MC. 1996. Platelet-streptococcal interactions in endocarditis. Crit Rev Oral Biol Med 7:222–236. doi:10.1177/10454411960070030201. 8909879

[11] Herzberg MC, MacFarlane GD, Gong K, Armstrong NN, Witt AR, Erickson PR, Meyer MW. 1992. The platelet interactivity phenotype of Streptococcus sanguis influences the course of experimental endocarditis. Infect Immun 60:4809–4818. doi:10.1128/iai.60.11.4809-4818.1992. 1398992 PMC258235

[12] Herzberg MC, Gong K, MacFarlane GD, Erickson PR, Soberay AH, Krebsbach PH, Manjula G, Schilling K, Bowen WH. 1990. Phenotypic characterization of Streptococcus sanguis virulence factors associated with bacterial endocarditis. Infect Immun 58:515–522. doi:10.1128/iai.58.2.515-522.1990. 2137112 PMC258487

[13] Dall LH, Herndon BL. 1990. Association of cell-adherent glycocalyx and endocarditis production by viridans group streptococci. J Clin Microbiol 28:1698–1700. doi:10.1128/jcm.28.8.1698-1700.1990. 2394799 PMC268030

[14] Baddour LM. 1994. Virulence factors among gram-positive bacteria in experimental endocarditis. Infect Immun 62:2143–2148. doi:10.1128/iai.62.6.2143-2148.1994. 8188334 PMC186490

[15] Nobbs AH, Lamont RJ, Jenkinson HF. 2009. Streptococcus adherence and colonization. Microbiol Mol Biol Rev 73:407–450. doi:10.1128/MMBR.00014-09. 19721085 PMC2738137

[16] Zheng W, Tan MF, Old LA, Paterson IC, Jakubovics NS, Choo SW. 2017. Distinct biological potential of Streptococcus gordonii and Streptococcus sanguinis revealed by comparative genome analysis. Sci Rep 7:2949. doi:10.1038/s41598-017-02399-4. 28592797 PMC5462765

[17] Dhotre S, Jahagirdar V, Suryawanshi N, Davane M, Patil R, Nagoba B. 2018. Assessment of periodontitis and its role in viridans streptococcal bacteremia and infective endocarditis. Indian Heart J 70:225–232. doi:10.1016/j.ihj.2017.06.019. 29716699 PMC5993913

[18] Douglas CW, Heath J, Hampton KK, Preston FE. 1993. Identity of viridans streptococci isolated from cases of infective endocarditis. J Med Microbiol 39:179–182. doi:10.1099/00222615-39-3-179. 8366515

[19] Horaud T, Delbos F. 1984. Viridans streptococci in infective endocarditis: species distribution and susceptibility to antibiotics. Eur Heart J 5(Suppl C):39–44. doi:10.1093/eurheartj/5.suppl_c.39. 6519085

[20] Henriksen SD, Henrichsen J. 1975. Twitching motility and possession of polar fimbriae in spreading Streptococcus sanguis isolates from the human throat. Acta Pathol Microbiol Scand B 83:133–140. doi:10.1111/j.1699-0463.1975.tb00083.x. 1171576

[21] Henriksen SD, Henrichsen J. 1976. Further studies of twitching Streptococcus sanguis isolated from the human throat. Isolation of strains with a new antigen. Acta Pathol Microbiol Scand B 84B:428–432. doi:10.1111/j.1699-0463.1976.tb01962.x. 826110

[22] Gaustad P, Frøholm LO. 1984. Genetic transformation in Streptococcus sanguis. Simultaneous variation of surface-spreading, competence, hemagglutination and polar fimbriation in selected strains. Acta Pathol Microbiol Immunol Scand B 92:283–289. doi:10.1111/j.1699-0463.1984.tb02835.x. 6152366

[23] Mattick JS. 2002. Type IV pili and twitching motility. Annu Rev Microbiol 56:289–314. doi:10.1146/annurev.micro.56.012302.160938. 12142488

[24] Pelicic V. 2008. Type IV pili: e pluribus unum? Mol Microbiol 68:827–837. doi:10.1111/j.1365-2958.2008.06197.x. 18399938

[25] Giltner CL, Nguyen Y, Burrows LL. 2012. Type IV pilin proteins: versatile molecular modules. Microbiol Mol Biol Rev 76:740–772. doi:10.1128/MMBR.00035-12. 23204365 PMC3510520

[26] Melville S, Craig L. 2013. Type IV pili in Gram-positive bacteria. Microbiol Mol Biol Rev 77:323–341. doi:10.1128/MMBR.00063-12. 24006467 PMC3811610

[27] Arlehamn CS, Evans TJ. 2011. Pseudomonas aeruginosa pilin activates the inflammasome. Cell Microbiol 13:388–401. doi:10.1111/j.1462-5822.2010.01541.x. 20955240 PMC3429865

[28] Bordeleau E, Purcell EB, Lafontaine DA, Fortier LC, Tamayo R, Burrus V. 2015. Cyclic di-GMP riboswitch-regulated type IV pili contribute to aggregation of Clostridium difficile. J Bacteriol 197:819–832. doi:10.1128/JB.02340-14. 25512308 PMC4325102

[29] Hendrick WA, Orr MW, Murray SR, Lee VT, Melville SB. 2017. Cyclic Di-GMP binding by an assembly ATPase (PilB2) and control of type IV pilin polymerization in the Gram-positive pathogen Clostridium perfringens. J Bacteriol 199:e00034-17. doi:10.1128/JB.00034-17. 28242722 PMC5405213

[30] Gurung I, Spielman I, Davies MR, Lala R, Gaustad P, Biais N, Pelicic V. 2016. Functional analysis of an unusual type IV pilus in the Gram-positive Streptococcus sanguinis. Mol Microbiol 99:380–392. doi:10.1111/mmi.13237. 26435398 PMC4832360

[31] Chen YM, Chiang YC, Tseng TY, Wu HY, Chen YY, Wu CH, Chiu CH. 2019. Molecular and functional analysis of the type IV pilus gene cluster in Streptococcus sanguinis SK36. Appl Environ Microbiol 85:e02788-18. doi:10.1128/AEM.02788-18. 30635384 PMC6414370

[32] Ota C, Morisaki H, Nakata M, Arimoto T, Fukamachi H, Kataoka H, Masuda Y, Suzuki N, Miyazaki T, Okahashi N, Kuwata H. 2018. Streptococcus sanguinis noncoding *cia*-dependent small RNAs negatively regulate expression of type IV pilus retraction ATPase PilT and biofilm formation. Infect Immun 86:e00894-17. doi:10.1128/IAI.00894-17. 29263111 PMC5820960

[33] Henriksen SD, Eriksen J. 1976. Transformation of twitching strains of Streptococcus sanguis. Acta Pathol Microbiol Scand B 84B:433–436. doi:10.1111/j.1699-0463.1976.tb01963.x. 998260

[34] Henriksen SD, Løvstad E. 1976. Haemagglutination of twitching Streptococcus sanguis. Acta Pathol Microbiol Scand B 84B:437–440. doi:10.1111/j.1699-0463.1976.tb01964.x. 998261

[35] Danne C, Dramsi S. 2012. Pili of gram-positive bacteria: roles in host colonization. Res Microbiol 163:645–658. doi:10.1016/j.resmic.2012.10.012. 23116627

[36] Varga JJ, Therit B, Melville SB. 2008. Type IV pili and the CcpA protein are needed for maximal biofilm formation by the gram-positive anaerobic pathogen Clostridium perfringens. Infect Immun 76:4944–4951. doi:10.1128/IAI.00692-08. 18765726 PMC2573335

[37] Rodgers K, Arvidson CG, Melville S. 2011. Expression of a Clostridium perfringens type IV pilin by Neisseria gonorrhoeae mediates adherence to muscle cells. Infect Immun 79:3096–3105. doi:10.1128/IAI.00909-10. 21646450 PMC3147591

[38] Martini AM, Moricz BS, Ripperger AK, Tran PM, Sharp ME, Forsythe AN, Kulhankova K, Salgado-Pabón W, Jones BD. 2020. Association of novel Streptococcus sanguinis virulence factors with pathogenesis in a native valve infective endocarditis model. Front Microbiol 11:10. doi:10.3389/fmicb.2020.00010. 32082276 PMC7005726

[39] Herrera A, Kulhankova K, Sonkar VK, Dayal S, Klingelhutz AJ, Salgado-Pabón W, Schlievert PM. 2017. Staphylococcal β-toxin modulates human aortic endothelial cell and platelet function through sphingomyelinase and biofilm ligase activities. mBio 8:e00273-17. doi:10.1128/mBio.00273-17. 28325766 PMC5362035

[40] White JC, Niven CF, Jr. 1946. Streptococcus S.B.E.: a streptococcus associated with subacute bacterial endocarditis. J Bacteriol 51:717–722. doi:10.1128/jb.51.6.717-722.1946. 16561124 PMC518116

[41] Durack DT, Beeson PB, Petersdorf RG. 1973. Experimental bacterial endocarditis. 3. Production and progress of the disease in rabbits. Br J Exp Pathol 54:142–151. 4700697 PMC2072580

[42] Durack DT. 1975. Experimental bacterial endocarditis. IV. Structure and evolution of very early lesions. J Pathol 115:81–89. doi:10.1002/path.1711150204. 1151519

[43] Werdan K, Dietz S, Löffler B, Niemann S, Bushnaq H, Silber RE, Peters G, Müller-Werdan U. 2014. Mechanisms of infective endocarditis: pathogen-host interaction and risk states. Nat Rev Cardiol 11:35–50. doi:10.1038/nrcardio.2013.174. 24247105

[44] Berry JL, Gurung I, Anonsen JH, Spielman I, Harper E, Hall AMJ, Goosens VJ, Raynaud C, Koomey M, Biais N, Matthews S, Pelicic V. 2019. Global biochemical and structural analysis of the type IV pilus from the Gram-positive bacterium Streptococcus sanguinis. J Biol Chem 294:6796–6808. doi:10.1074/jbc.RA118.006917. 30837269 PMC6497953

[45] Konto-Ghiorghi Y, Mairey E, Mallet A, Duménil G, Caliot E, Trieu-Cuot P, Dramsi S. 2009. Dual role for pilus in adherence to epithelial cells and biofilm formation in Streptococcus agalactiae. PLoS Pathog 5:e1000422. doi:10.1371/journal.ppat.1000422. 19424490 PMC2674936

[46] Nielsen HV, Guiton PS, Kline KA, Port GC, Pinkner JS, Neiers F, Normark S, Henriques-Normark B, Caparon MG, Hultgren SJ. 2012. The metal ion-dependent adhesion site motif of the Enterococcus faecalis EbpA pilin mediates pilus function in catheter-associated urinary tract infection. mBio 3:e00177-12. doi:10.1128/mBio.00177-12. 22829678 PMC3419518

[47] Morgan J, Saleem M, Ng R, Armstrong C, Wong SS, Caulton SG, Fickling A, Williams HEL, Munday AD, López JA, Searle MS, Emsley J. 2019. Structural basis of the leukocyte integrin Mac-1 I-domain interactions with the platelet glycoprotein Ib. Blood Adv 3:1450–1459. doi:10.1182/bloodadvances.2018027011. 31053572 PMC6517656

[48] Berry JL, Pelicic V. 2015. Exceptionally widespread nanomachines composed of type IV pilins: the prokaryotic Swiss Army knives. FEMS Microbiol Rev 39:134–154. doi:10.1093/femsre/fuu001. 25793961 PMC4471445

[49] Leuck AM, Johnson JR, Dunny GM. 2014. A widely used in vitro biofilm assay has questionable clinical significance for enterococcal endocarditis. PLoS One 9:e107282. doi:10.1371/journal.pone.0107282. 25255085 PMC4177788

[50] Ge X, Kitten T, Chen Z, Lee SP, Munro CL, Xu P. 2008. Identification of Streptococcus sanguinis genes required for biofilm formation and examination of their role in endocarditis virulence. Infect Immun 76:2551–2559. doi:10.1128/IAI.00338-08. 18390999 PMC2423065

[51] Jung CJ, Yeh CY, Shun CT, Hsu RB, Cheng HW, Lin CS, Chia JS. 2012. Platelets enhance biofilm formation and resistance of endocarditis-inducing streptococci on the injured heart valve. J Infect Dis 205:1066–1075. doi:10.1093/infdis/jis021. 22357661

[52] Elgharably H, Hussain ST, Shrestha NK, Blackstone EH, Pettersson GB. 2016. Current hypotheses in cardiac surgery: biofilm in infective endocarditis. Semin Thorac Cardiovasc Surg 28:56–59. doi:10.1053/j.semtcvs.2015.12.005. 27568136

[53] Hall-Stoodley L, Costerton JW, Stoodley P. 2004. Bacterial biofilms: from the natural environment to infectious diseases. Nat Rev Microbiol 2:95–108. doi:10.1038/nrmicro821. 15040259

[54] Salgado-Pabón W, Schlievert PM. 2015. Aortic valve damage for the study of left-sided native valve infective endocarditis in rabbits, p 73–80. *In* Superantigens. Springer, New York, NY. 10.1007/978-1-4939-3344-0_6 26676038

[55] Pelicic V. 2019. Monoderm bacteria: the new frontier for type IV pilus biology. Mol Microbiol 112:1674–1683. doi:10.1111/mmi.14397. 31556183 PMC6916266

[56] Nagata E, de Toledo A, Oho T. 2011. Invasion of human aortic endothelial cells by oral viridans group streptococci and induction of inflammatory cytokine production. Mol Oral Microbiol 26:78–88. doi:10.1111/j.2041-1014.2010.00597.x. 21214874

[57] van der Meijden PEJ, Heemskerk JWM. 2019. Platelet biology and functions: new concepts and clinical perspectives. Nat Rev Cardiol 16:166–179. doi:10.1038/s41569-018-0110-0. 30429532

[58] López JA. 2017. The platelet glycoprotein Ib-IX-V complex, p 85–97. *In* Platelets in thrombotic and non-thrombotic disorders. Springer International Publishing, New York, NY.

[59] Johnson CM, Bowie EJ. 1992. Pigs with von Willebrand disease may be resistant to experimental infective endocarditis. J Lab Clin Med 120:553–558. 1402331

[60] Liesenborghs L, Meyers S, Vanassche T, Verhamme P. 2020. Coagulation: at the heart of infective endocarditis. J Thromb Haemost 18:995–1008. doi:10.1111/jth.14736. 31925863

[61] Yuan H, Deng N, Zhang S, Cao Y, Wang Q, Liu X, Zhang Q. 2012. The unfolded von Willebrand factor response in bloodstream: the self-association perspective. J Hematol Oncol 5:65. doi:10.1186/1756-8722-5-65. 23067373 PMC3488313

[62] Sakariassen KS, Bolhuis PA, Sixma JJ. 1979. Human blood platelet adhesion to artery subendothelium is mediated by factor VIII-Von Willebrand factor bound to the subendothelium. Nature 279:636–638. doi:10.1038/279636a0. 313016

[63] Goldsmith HL, Turitto VT. 1986. Rheological aspects of thrombosis and haemostasis: basic principles and applications. ICTH-Report–Subcommittee on Rheology of the International Committee on Thrombosis and Haemostasis. Thromb Haemost 55:415–435. doi:10.1055/s-0038-1661576. 3750272

[64] Bensing BA, Loukachevitch LV, McCulloch KM, Yu H, Vann KR, Wawrzak Z, Anderson S, Chen X, Sullam PM, Iverson TM. 2016. Structural basis for sialoglycan binding by the Streptococcus sanguinis SrpA adhesin. J Biol Chem 291:7230–7240. doi:10.1074/jbc.M115.701425. 26833566 PMC4817157

[65] Plummer C, Wu H, Kerrigan SW, Meade G, Cox D, Ian DCW. 2005. A serine-rich glycoprotein of Streptococcus sanguis mediates adhesion to platelets via GPIb. Br J Haematol 129:101–109. doi:10.1111/j.1365-2141.2005.05421.x. 15801962

[66] Raynaud C, Sheppard D, Berry JL, Gurung I, Pelicic V. 2021. PilB from *Streptococcus sanguinis* is a bimodular type IV pilin with a direct role in adhesion. Proc Natl Acad Sci USA 118:e2102092118. doi:10.1073/pnas.2102092118. 34031252 PMC8179133

[67] Puccio T, Kunka KS, Zhu B, Xu P, Kitten T. 2020. Manganese depletion leads to multisystem changes in the transcriptome of the opportunistic pathogen Streptococcus sanguinis. Front Microbiol 11:592615. doi:10.3389/fmicb.2020.592615. 33250881 PMC7674665

[68] Crump KE, Bainbridge B, Brusko S, Turner LS, Ge X, Stone V, Xu P, Kitten T. 2014. The relationship of the lipoprotein SsaB, manganese and superoxide dismutase in Streptococcus sanguinis virulence for endocarditis. Mol Microbiol 92:1243–1259. doi:10.1111/mmi.12625. 24750294 PMC4070010

[69] Meyer MW, Gong K, Herzberg MC. 1998. Streptococcus sanguis-induced platelet clotting in rabbits and hemodynamic and cardiopulmonary consequences. Infect Immun 66:5906–5914. doi:10.1128/IAI.66.12.5906-5914.1998. 9826372 PMC108748

[70] Lowrance JH, Baddour LM, Simpson WA. 1990. The role of fibronectin binding in the rat model of experimental endocarditis caused by Streptococcus sanguis. J Clin Invest 86:7–13. doi:10.1172/JCI114717. PMC296682 2164050

[71] Pulliam L, Dall L, Inokuchi S, Wilson W, Hadley WK, Mills J. 1985. Effects of exopolysaccharide production by viridans streptococci on penicillin therapy of experimental endocarditis. J Infect Dis 151:153–156. doi:10.1093/infdis/151.1.153. 3965587

[72] Zhu B, Macleod LC, Kitten T, Xu P. 2018. Streptococcus sanguinis biofilm formation & interaction with oral pathogens. Future Microbiol 13:915–932. doi:10.2217/fmb-2018-0043. 29882414 PMC6060398

[73] Rhodes DV, Crump KE, Makhlynets O, Snyder M, Ge X, Xu P, Stubbe J, Kitten T. 2014. Genetic characterization and role in virulence of the ribonucleotide reductases of Streptococcus sanguinis. J Biol Chem 289:6273–6287. doi:10.1074/jbc.M113.533620. 24381171 PMC3937693

[74] Paik S, Senty L, Das S, Noe JC, Munro CL, Kitten T. 2005. Identification of virulence determinants for endocarditis in Streptococcus sanguinis by signature-tagged mutagenesis. Infect Immun 73:6064–6074. doi:10.1128/IAI.73.9.6064-6074.2005. 16113327 PMC1231064

[75] Deng L, Bensing BA, Thamadilok S, Yu H, Lau K, Chen X, Ruhl S, Sullam PM, Varki A. 2014. Oral streptococci utilize a Siglec-like domain of serine-rich repeat adhesins to preferentially target platelet sialoglycans in human blood. PLoS Pathog 10:e1004540. doi:10.1371/journal.ppat.1004540. 25474103 PMC4256463

[76] Fan J, Zhang Y, Chuang-Smith ON, Frank KL, Guenther BD, Kern M, Schlievert PM, Herzberg MC. 2012. Ecto-5’-nucleotidase: a candidate virulence factor in Streptococcus sanguinis experimental endocarditis. PLoS One 7:e38059. doi:10.1371/journal.pone.0038059. 22685551 PMC3369921

[77] Xu P, Alves JM, Kitten T, Brown A, Chen Z, Ozaki LS, Manque P, Ge X, Serrano MG, Puiu D, Hendricks S, Wang Y, Chaplin MD, Akan D, Paik S, Peterson DL, Macrina FL, Buck GA. 2007. Genome of the opportunistic pathogen Streptococcus sanguinis. J Bacteriol 189:3166–3175. doi:10.1128/JB.01808-06. 17277061 PMC1855836

[78] Gurung I, Berry JL, Hall AMJ, Pelicic V. 2017. Cloning-independent markerless gene editing in Streptococcus sanguinis: novel insights in type IV pilus biology. Nucleic Acids Res 45:e40. doi:10.1093/nar/gkw1177. 27903891 PMC5389465

[79] You C, Zhang YH. 2014. Simple cloning and DNA assembly in Escherichia coli by prolonged overlap extension PCR. Methods Mol Biol 1116:183–192. doi:10.1007/978-1-62703-764-8_13. 24395365

[80] García-Nafría J, Watson JF, Greger IH. 2016. IVA cloning: a single-tube universal cloning system exploiting bacterial in vivo assembly. Sci Rep 6:27459. doi:10.1038/srep27459. 27264908 PMC4893743

[81] Dyballa N, Metzger S. 2012. Fast and sensitive Coomassie staining in quantitative proteomics, p 47–59. *In* Marcus K (ed), Quantitative methods in proteomics. Humana Press, Totowa, NJ. 10.1007/978-1-61779-885-6_4 22665293

[82] Stinson MW, Alder S, Kumar S. 2003. Invasion and killing of human endothelial cells by viridans group streptococci. Infect Immun 71:2365–2372. doi:10.1128/IAI.71.5.2365-2372.2003. 12704106 PMC153257

