# Supplementary material for: Type IV Pili of Streptococcus sanguinis Contribute to Pathogenesis in Experimental Infective Endocarditis
Source: Microbiol Spectr. 2021 Nov 10;9(3):e01752-21. doi: 10.1128/Spectrum.01752-21 (PMC8579931; doi:10.1128/Spectrum.01752-21)
Supplement: SUPPLEMENTAL FILE 1 — Supplemental material. Download SPECTRUM01752-21_Supp_1_seq13.pdf, PDF file, 2.9 MB [file spectrum01752-21_supp_1_seq13.pdf]

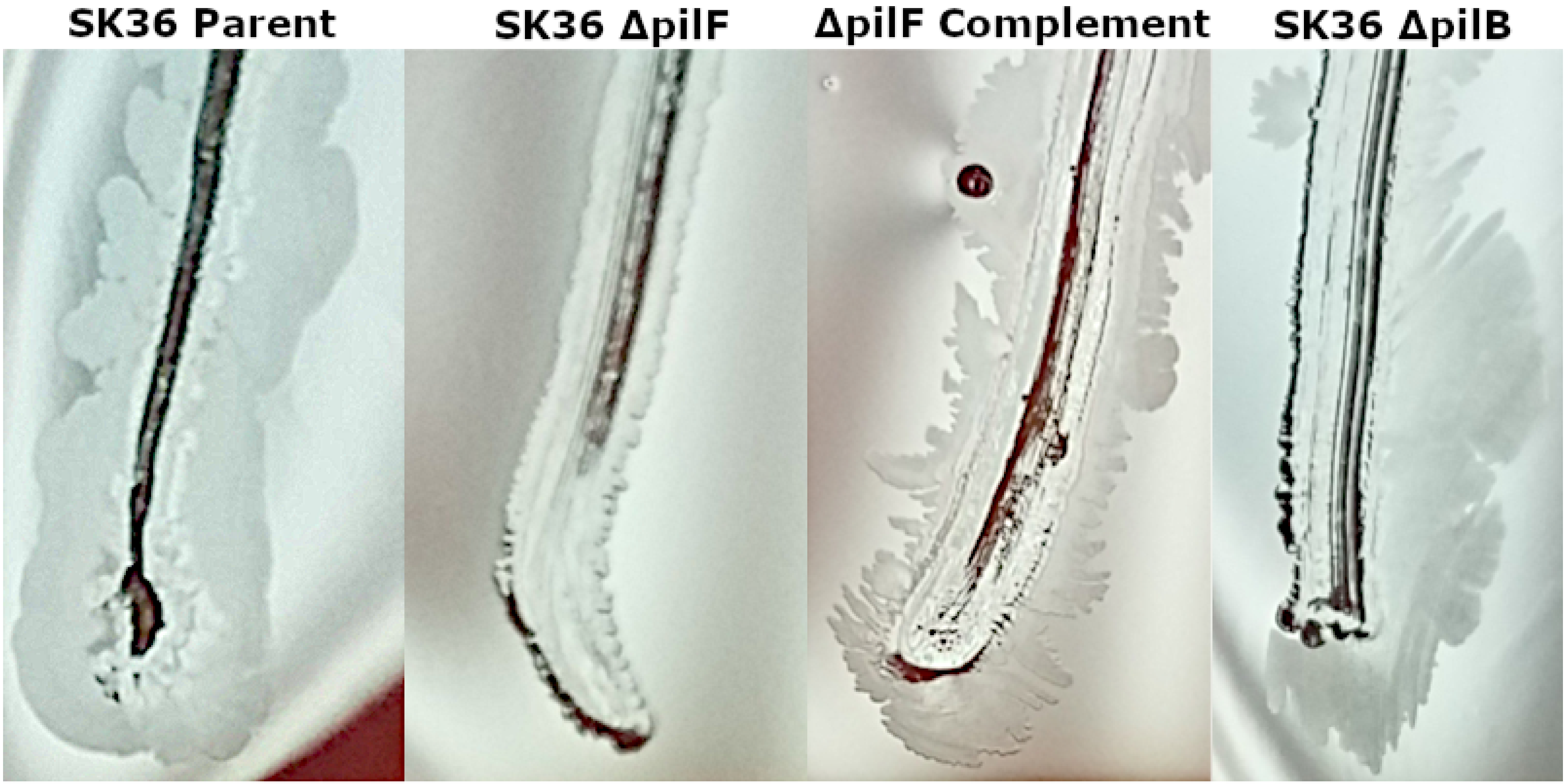
SK36 Parent
SK36 ΔpilF
ΔpilF Complement
SK36 ΔpilB

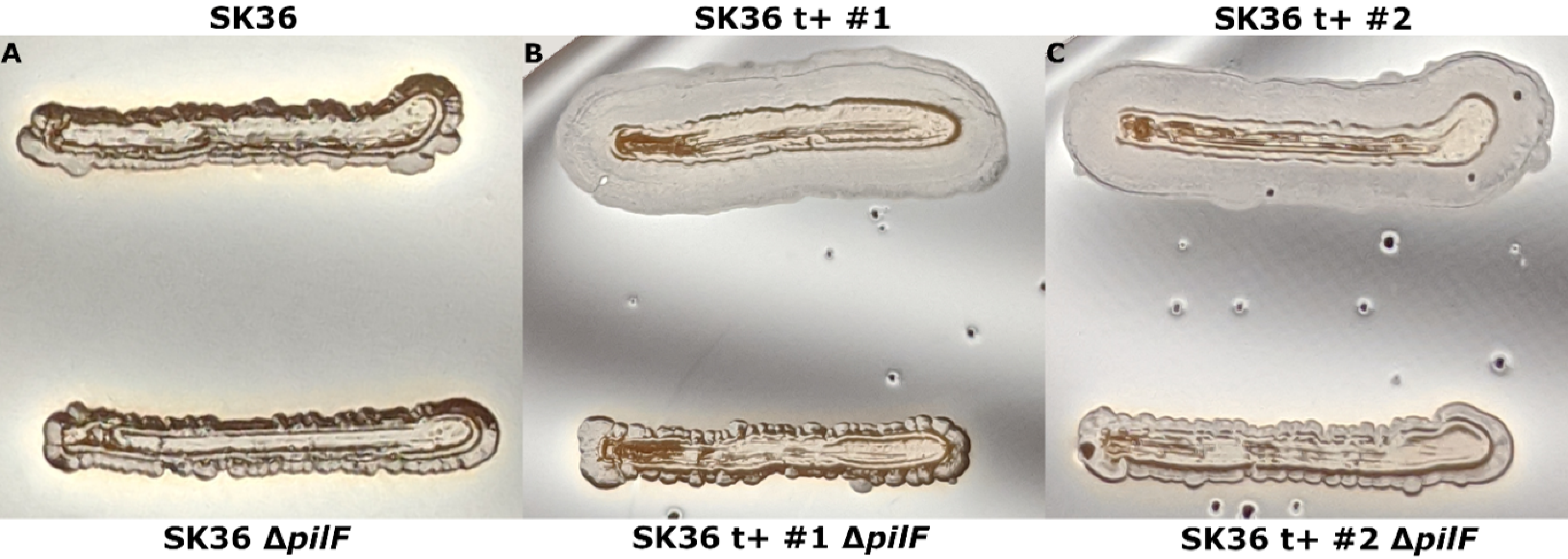
SK36
SK36 t+ #1
SK36 t+ #2
A
B
C
SK36 Δ*pilF*
SK36 t+ #1 Δ*pilF*
SK36 t+ #2 Δ*pilF*

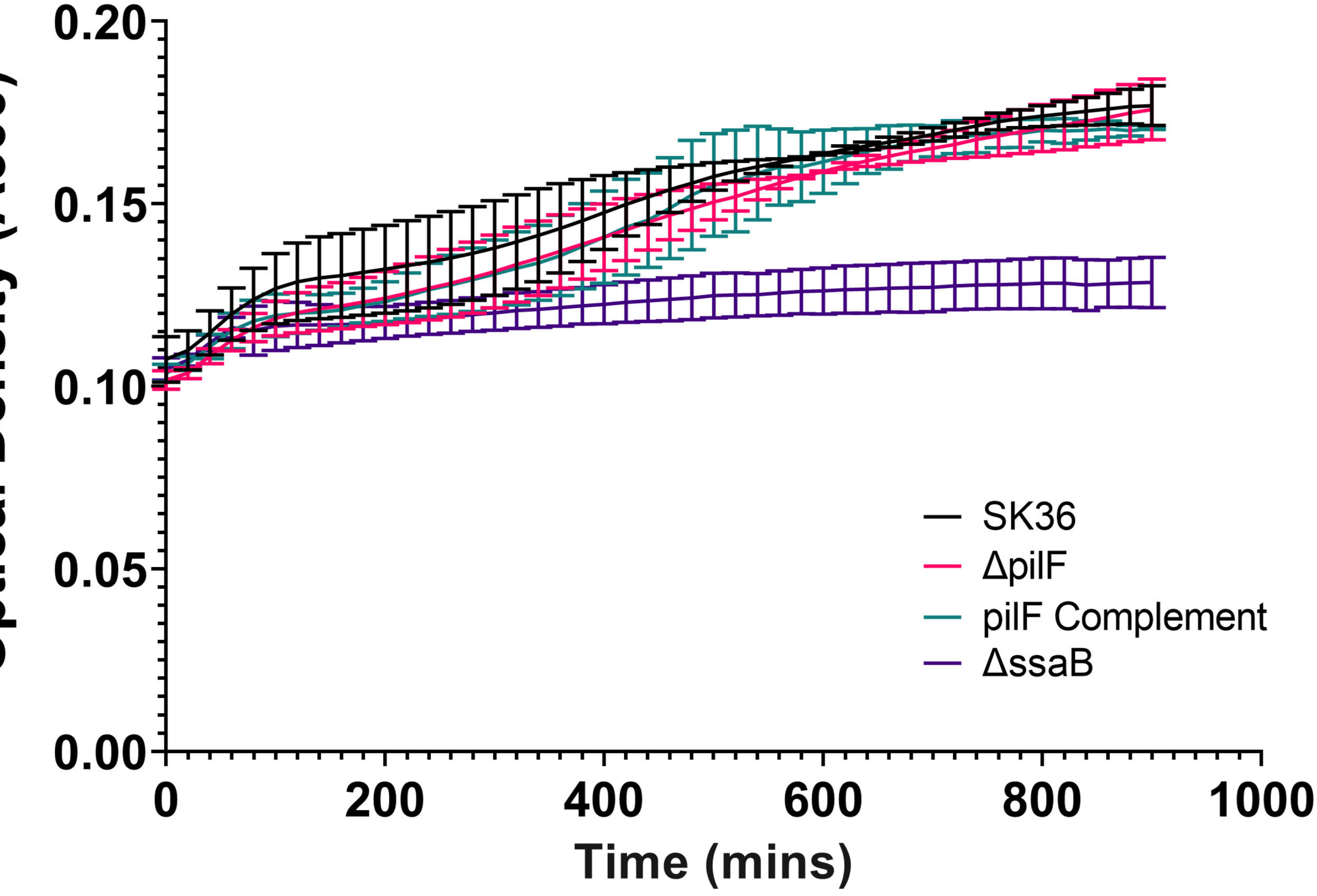
0.20
0.15
0.10
0.05
0.00
0
200
400
600
800
1000
Time (mins)
SK36
ΔpilF
pilF Complement
ΔssaB

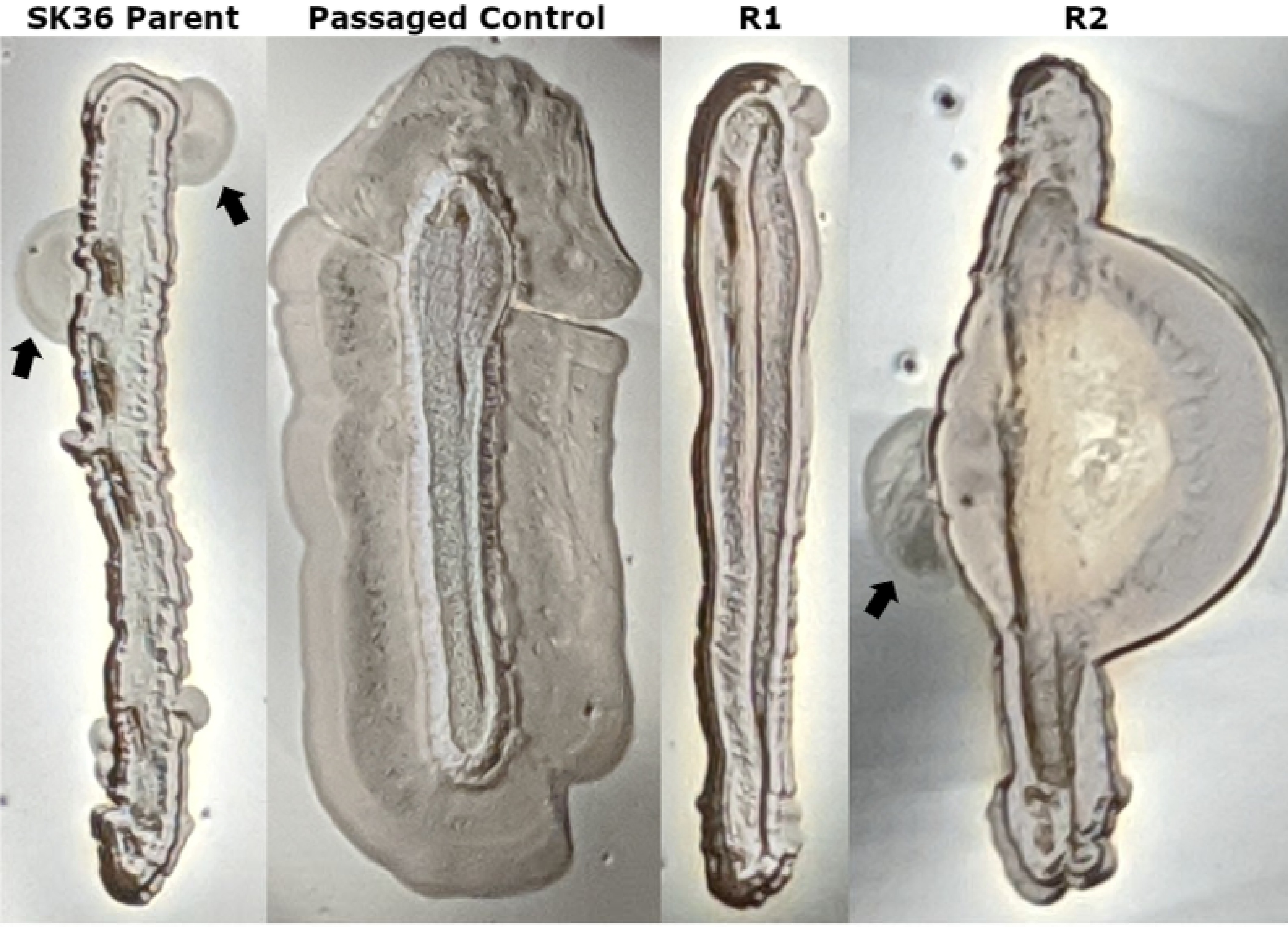
SK36 Parent
Passaged Control
R1
R2

Supplementary Material

Supplemental Figure 1: The SK36 Δ*pilF* complement strain develops twitching motility during growth on blood agar as seen for the SK36 parent strain. The Δ*pilF* mutant fails to exhibit any twitching motility even after multiple passages (not shown).

Supplemental Figure 2: Twitching phenotypes of passaged S. sanguinis SK36 and SK36 Δ*pilF*. Images were taken on day 4 of the first passage. A) SK36 does not exhibit twitching motility following first passage on blood agar. B & C) Individual twitching colonies isolated previously ("t+" designation) are capable of twitching motility following first passage (top) but lose this ability when the Δ*pilF* mutation is introduced.

Supplemental Figure 3: SK36 WT, Δ*pilF* mutant, and *pilF* complement exhibit similar growth in 100% rabbit serum. A Δ*ssaB* strain is included as a control. Statistical analysis was performed using two-way ANOVA and corrected for multiple comparisons by the method of Dunnett. $* = p < 0.05$. Error bars represent the 95% confidence intervals. Growth kinetics of the Δ*ssaB* mutant reach statistical significance compared to WT starting at 400 minutes.

Supplemental Figure 4: S. sanguinis SK36 isolated from two different WT-infected rabbits (R1 and R2) were assayed for their twitching motility phenotype following infection. As shown, SK36 following infection exhibits a motility phenotype similar to the SK36 parent rather than the passaged, hyper-twitching control strains. Arrows identify nascent zones of twitching in both the parent and R2 populations.
